# Activation mechanism of the full-length histidine kinase LvrB from pathogenic *Leptospira*

**DOI:** 10.1038/s41467-026-71783-4

**Published:** 2026-04-16

**Authors:** Elia Agustoni, Ariel Mechaly, Joaquín Dalla Rizza, David Beriashvili, Kristyna Pluhackova, Polina Isaikina, Felipe Trajtenberg, Thomas Müntener, Elsio A. Wunder, Albert I. Ko, Tilman Schirmer, Alejandro Buschiazzo, Sebastian Hiller

**Affiliations:** 1 Biozentrum, University of Basel, Basel, Switzerland; 2 Institut Pasteur de Montevideo, Montevideo, Uruguay; 3 Institut Pasteur, Paris, France; 4 Stuttgart Center for Simulation Science, Cluster of Excellence EXC 2075, University of Stuttgart, Stuttgart, Germany; 5 Yale School of Public Health, New Haven, CT USA; 6 University of Connecticut, Storrs, CT USA; 7 Instituto Gonçalo Moniz, Fundação Oswaldo Cruz/MS, Salvador, Brazil; 8 Present Address: Center for Life Sciences, Paul Scherrer Institute, Villigen, Switzerland

**Keywords:** Cryoelectron microscopy, Bacterial structural biology, Kinases

## Abstract

Pathogenic *Leptospira* modulate their virulence via the Lvr signaling system, with the histidine kinase LvrB being a central element. LvrB is a prototype of Rec-controlled histidine kinases, which are frequently found in bacterial two-component systems, and yet whose regulatory mechanisms remain largely unknown. Here, we report full-length structures of LvrB in different states uncovering its mechanism of activation. Kinase-inactive LvrB is a symmetric homodimer, with its catalytic domains rigidly clasped onto the central helical domain. Phosphorylation of the N-terminal Rec domains induces coiled-coil formation of the central αS helices thereby breaking symmetry through liberation of the catalytic domains into a dynamic, auto-phosphorylation competent state. We further identified LvrB’s downstream effector partner LvrC, an anti-σ factor that reprograms the transcription of hundreds of virulence genes. Our findings set a mechanistic paradigm for Rec-controlled histidine kinases enabling the design of virulence inhibitors.

## Abstract (web summary)

The study reveals how the bacterial histidine kinase LvrB is activated: phosphorylation triggers coiled-coil formation, symmetry breaking and catalytic domain release, resulting in virulence gene expression.

## Introduction

Leptospirosis is a widespread zoonosis and severe public health problem in resource-poor settings that is showing epidemiological reemergence with climate change^1,2^. The disease can provoke life-threatening symptoms, including vascular injury, organ failure, and pulmonary hemorrhage^3,4^. Virulence in highly pathogenic *Leptospira* species is modulated by the Leptospira virulence regulator (Lvr) phosphorelay pathway^5,6^, which relies on the histidine kinase (HK) LvrB as a key component. LvrB is activated by the upstream HK LvrA, and, in turn, controls a yet unknown downstream factor to ultimately reprogram the expression of hundreds of genes^7^.

**Fig. 1:**
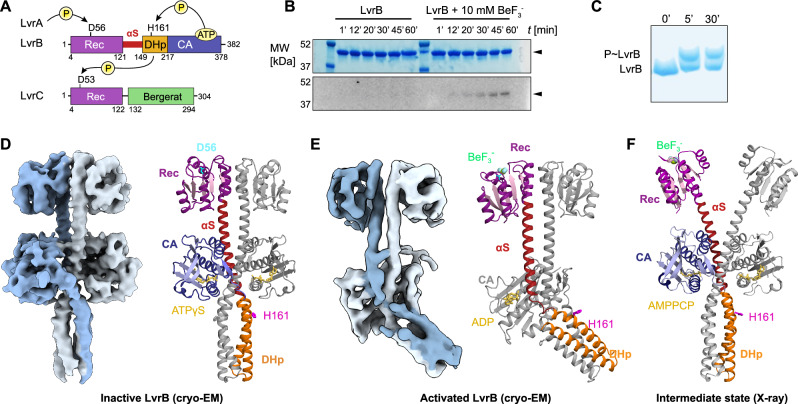
Structures of activated and inactive LvrB. **A** Lvr signaling pathway with autophosphorylation and phosphotransfer reactions highlighted by arrows. **B** Time-dependent LvrB autophosphorylation in the absence and presence of beryllofluoride, detected by γ−^32^P-ATP autoradiography. The experiment has been performed *N* = 3 times with similar results. **C** Phos-Tag acrylamide gel, suggesting that the LvrB dimer undergoes hemi-phosphorylation. The experiment has been performed *N* = 3 times with similar results. **D** Sharpened cryo-EM map of the LvrB homodimer at 4.2 Å and atomic model refined within the map. Domain coloring scheme of one protomer, as in (**A**), and the other protomer in gray. The phosphorylatable residues D56 and H161 are colored in cyan and magenta, respectively. **E** Sharpened cryo-EM map of (pseudo)phosphorylated LvrB:BeF_3_^−^ homodimer in solution at 5.9 Å and atomic model refined within the map. One CA domain is seen packed against the central helical bundle, with the other not yielding coherent density. **F** Crystal structure of LvrB:BeF_3_^−^. The structure is similar to the inactive structure, but with the Rec domains splayed apart.

HKs regulate vital processes in microorganisms and plants as part of two-component systems (TCS) and phosphorelays. HKs sense external and internal signals, regulating their own phosphorylation. The phosphoryl group is then transferred onto the receiver (Rec) domain of a cognate response regulator (RR)^8^. The HK core, comprised of an α-helical DHp (Dimerization and Histidine phosphotransfer) and a CA (Catalytic ATP-binding) domain, is sufficient for the phosphoryl-transfer catalysis^9–11^. In most HKs, the activity of this HK core is controlled by N-terminal sensory domains. Structure and function of the HK core and sensory domains have been thoroughly studied in isolation; however, little is known about their interplay in a full-length context^9,12^. Conspicuously, in many of these dimeric enzymes, the input domain and HK core are joined by a helical linker with coiled-coil propensity, the αS helix, which appears to be central for allosteric control^13,14^. Various changes in coiled-coil geometry upon signal perception have been proposed, including piston, scissor, flip-flop, or rotational movements^11,14–17^, but thus far none have been observed directly in a full-length histidine kinase. LvrB features a singular Rec–DHp–CA domain architecture (Fig. 1A), making it both a histidine kinase and a response regulator. Hence, LvrB is a prototype for Rec-controlled HKs, utilizing Rec phosphorylation as the sensory input signal^18,19^. Studies of LvrB are thus not only expected to elucidate a central mechanism of *Leptospira* virulence control, but also to unravel the mechanistic basis of αS-mediated HK activation.

Here, we report full-length structures of LvrB in the inactive and activated states, discerning the allosteric activation mechanism. Pseudo-phosphorylation of the sensory Rec domain induces αS coiled-coil formation that is concomitant with a break of the overall symmetry and liberation of the CA domains from their tightly bound configuration. Furthermore, we identify LvrB’s specific downstream response regulator, which we name LvrC. Its crystal structure reveals that LvrC comprises a Rec domain joined to an anti-σ factor output effector domain, which readily explains the global scale of transcriptional reprogramming induced by the Lvr pathway during infection^7^.

## Results

### Structures of LvrB in different states uncover the activation mechanism

Recombinant full-length LvrB was purified to homogeneity. The protein was fully functional as it formed homodimers that bound ATP, and could be activated by the phosphorylation mimetic beryllofluoride (BeF_3_^−^; Fig. 1B and Supplementary Fig. 1)^20,21^. Phos-tag acrylamide gel electrophoresis suggested that active LvrB hemi-phosphorylates, i.e., only one DHp subunit undergoes autophosphorylation at a time, as many HKs do (Fig. 1C)^10^.

The structure of unphosphorylated full-length LvrB with bound ATPγS and Mg^2+^ was resolved to 4.2 Å resolution by cryo-EM (Fig. 1D and Supplementary Figs. 2 and 3, Supplementary Table 1). In this preparation, the non-hydrolysable ATP-mimic ATPγS was used to capture the CA domains in a physiologically relevant state. Given that ATP is present at high concentrations within cells, it is expected that the CA domain ATP-binding pocket is generally loaded with a nucleotide. The homodimer shows overall 2-fold symmetry, with the major inter-subunit contact mediated by the canonical 4-helix bundle of the DHp domain (Fig. 1D). Each Rec domain is connected to the DHp domain by a single, long and uninterrupted helix which fuses helix α5 of the Rec domain, the αS helix, and DHp helix α1′. Despite their high coiled-coil propensity (Supplementary Fig. 4), the two αS helices do not interact, as they are splayed apart by the Rec domains at their N-terminal ends. The CA domains are attached laterally to the αS/DHp region of the opposing subunit, with their helices α1 and α2 forming hydrophobic contacts with αS residues L139 and L143, and the “thumb” F330 and the “gripper helix” α3 interacting with DHp α1 (Supplementary Fig. 3H, I). Clearly, in this conformation, the CA domains are not productive for auto-phosphorylation, due to an ATP–H161 distance >20 Å, consistent with the observed low enzymatic activity (Fig. 1B).

The cryo-EM density of activated LvrB was obtained upon incubation with the phosphoryl-mimic BeF_3_^−^ and in conjunction with ADP and Mg^2+^ (Fig. 1E and Supplementary Fig. 5, Supplementary Table 1). BeF_3_^−^ was used to mimic the phosphorylation chemistry of the receiving aspartate D56^22^, and ADP was chosen to create the post-hydrolysis state. Despite the overall limited resolution of 5.9 Å, likely caused by pronounced particle asymmetry and anisotropic orientation, unambiguous placement of the individual domains was nevertheless possible. Strikingly, in this structure, the Rec domains appear rearranged, and the two αS helices are in direct contact, forming a regular left-handed coiled coil. The overall symmetry of the dimer is broken, as the central helical bundle features a pronounced kink of ~40° at the αS/DHp junction. One of the two CA domains exhibited a well-defined cryo-EM density, whereas the other only gave rise to a diffuse signal, suggesting that it rapidly samples a large conformational space (Fig. 1E and Supplementary Fig. 5). As in the inactive structure, the localized CA domain is bound laterally to the αS/DHp junction, but this time to residues of the same protomer and in a distinct orientation.

To enhance the interpretation of the cryo-EM densities, full-length LvrB was crystallized in complex with BeF_3_^−^, the ATP-mimic β,γ-methyleneadenosine-5′-triphosphate (AMP-PCP), and Mg^2+^, and the structure was solved to 2.6 Å resolution by X-ray diffraction (Fig. 1F and Supplementary Fig. 6, Supplementary Table 2). The protein crystallized in a conformation that was different from the activated and the inactive cryo-EM structures. Crystal packing led to pairs of interlocked LvrB dimers with the αS helices splayed widely apart, adopting a tetrameric conformation that is not observed in solution (Supplementary Figs. 1B, F and 6A). Though this tetramer is non-physiological, the individual dimer conformation must be transiently sampled and may thus resemble an intermediate state on the way to kinase activation. The high resolution provided reliable side chain orientations, including the confirmation that BeF_3_^−^ had properly mimicked Asp phosphorylation. Furthermore, two mechanistic insights were uncovered. Firstly, the CA domains were found in the same conformation as in the inactive state, thus establishing that splaying of the helices is directly connected to fastening the CA domains onto the central DHp. Secondly, Rec dimerization was found to be intrinsically weak, yet essential for coiled-coil formation, symmetry breaking, and thus LvrB activation. Overall, the set of two cryo-EM structures and one high-resolution crystal structure proved sufficient to elucidate the activation mechanism, as discussed below.

### Structural rearrangement of Rec domains

Many Rec domains possess a “Y-T switch” which serves to connect input phosphorylation with structural rearrangements^8,23^. In Y-T switches, an aromatic Y/F residue and a polar T/S residue are located close to the phosphorylatable site, in a loose packing that permits side chain rotation^24^. In the unphosphorylated state, these side chains appear spatially uncontrolled and can therefore point either inwards or outwards. Phosphorylation of the aspartate, however, pulls the T/S and the Y/F side chain strictly inwards towards the phosphorylated site (Supplementary Fig. 7A).

**Fig. 2:**
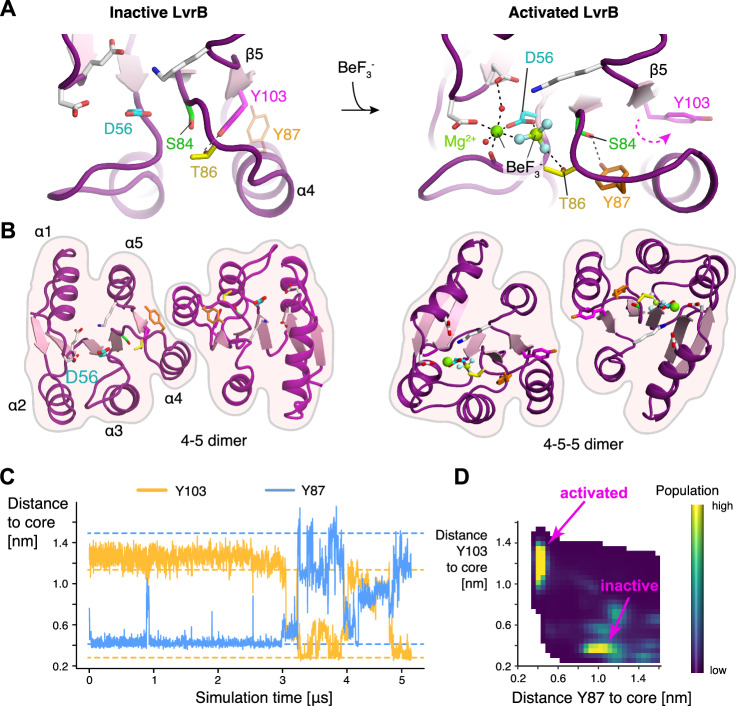
Rec domain rearrangement upon LvrB activation. **A** Structural details of the inverse Y-T switch. Left: unphosphorylated cryo-EM structure. Right: pseudo-phosphorylated crystal structure of LvrB:AMP-PCP:BeF_3_^−^. (Pseudo)phosphorylation pushes Y103 to the outward orientation, while pulling Y87 inside. **B** Top view of LvrB in its inactive and activated form, with a top-down view of the two different Rec dimers. **C** MD simulations of Y87/Y103 exchange within an isolated Rec domain. The simulations were initiated from activated phosphorylated Rec (Y103 outward, Y87 inward). The dashed horizontal lines indicate the experimental distances for the two states. **D** 2D population histogram of Y103 and Y87 relative to the inside of the Rec domain from *N* = 6 MD simulations (*n* = 3 per condition, 5 µs each, no data excluded). The two yellow maxima highlighted by magenta arrows show that Y103 and Y87 in the Rec core are anticorrelated. The two maxima correspond to the two experimentally observed states, as indicated.

In LvrB, the Y-T switch is expected to be formed by residues Y103 and S84, which are in the canonical positions on β5 and β4, respectively. In the unphosphorylated state, Y103 adopts an inward-facing position in a hydrophobic pocket formed by α4 and β4, and helices α5 and α5′ are far apart (Fig. 2A, B). Strikingly, however, in the pseudo-phosphorylated state, the side chain of Y103 is oriented outwards, and the α5 helices are moved towards each other. This happens because LvrB features an additional threonine residue near the Y-T switch, namely T86. This additional threonine coordinates the phosphate instead of the canonical S84, which in turn hydrogen-bonds to Y87, pulling helix α4 towards the phosphorylation center and pushing Y103 out. The Y-T switch in LvrB thus behaves exactly opposite to the canonical mechanism; it is an “inverse Y-T switch.”

We investigated the dynamics of this inverse Y-T switch by all-atom molecular dynamics (MD) simulations. The dynamics of an isolated Rec domain was simulated starting from either the unphosphorylated inactive conformation or the activated, phosphorylated conformation. In the absence of the lateral pressure from the second Rec domain, these simulations probe whether the inwards/outwards flips of the key residues Y87 and Y103 are coupled. Strikingly, spontaneous flips of these residues were observed on the microsecond timescale, and these were indeed anticorrelated, i.e., Y103 and Y87 alternated between inward and outward orientations (Fig. 2C, D and Supplementary Fig. 8A). These simulations thus support our mechanistic interpretation of the inverse Y-T switch.

In full-length LvrB, the side chain flips of the inverse Y-T switch result in a rearrangement of the Rec domain dimer, from the 4-5 dimer to the 4-5-5 dimer. Transitions between these two arrangements have been previously observed for Rec domains of the NtrC subfamily, such as DctD and NtrC (Fig. 2B and Supplementary Figs. 7B–D and 8B)^8^. Intriguingly, because the Y-T switch of LvrB is inverse, the switch between the 4-5 and 4-5-5 arrangements is also inverted relative to the Rec domains. In the non-phosphorylated state of LvrB, the Rec domains dimerize via the α4-β5 interface with a buried surface area of ~390 Å^2^. In contrast, in the (pseudo)-phosphorylated state, the LvrB Rec domains dimerize via the larger α4-β5-α5 interface with ~600 Å^2^ buried surface area.

### αS coiled-coil formation upon activation

**Fig. 3:**
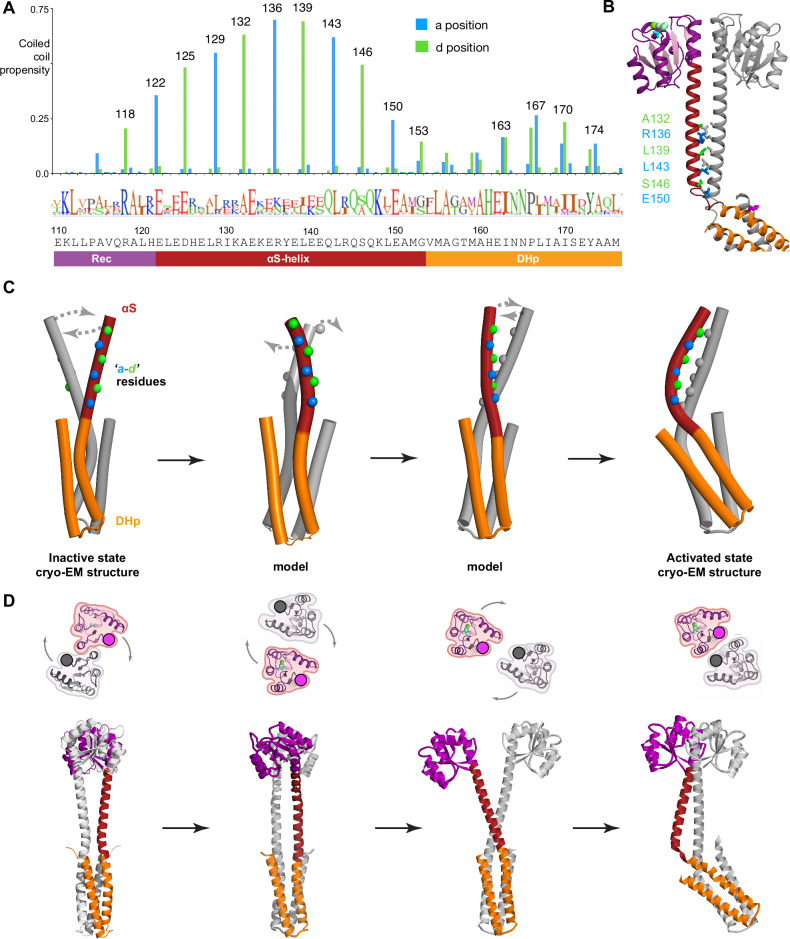
Coiled-coil formation and symmetry breaking upon LvrB activation. **A** Sequence analysis of LvrB(110–176), with coiled-coil propensity of the individual residues as determined by deepcoil2. **B** Close-up of the coiled coil in activated LvrB, with selected residues highlighted. Residue E150 appears to break the coiled coil. **C** Large-scale conformational changes of LvrB induced by (pseudo)phosphorylation. The αS helix and the DHp bundle are shown as cylinders; the CA domains and Rec domains have been omitted for clarity. The six *a*–*d* residues 129, 132, 136, 139, 143, and 146 are shown as green and blue spheres. The inactive and activated states have been determined experimentally, and the two conformations in between have been tentatively modeled. **D** Same as (**C**), with rearrangement of the Rec domains shown from the top and movement of Rec–αS–DHp from the side. Note that supercoiling changes from right- to left-handed.

As the next step of the signaling mechanism, the Rec dimer arrangement is coupled to the coiled-coil formation of the αS helices. The long signaling helix αS is the direct continuation of the Rec α5. The αS helix contains three coiled-coil heptad repeats in the 21-residue span from R129 to L149. Each heptad repeat features two residues, *a* and *d*, destined to form the interface of a parallel coiled coil with the other αS helix (Fig. 3A and Supplementary Fig. 4A)^25,26^.

Towards the C-terminus, the αS helices extend directly into the DHp bundle, specifically the pair of DHp α1 helices, which also comprise a coiled coil. Importantly, the heptad repeats of αS helix and α1 are separated by a formal 3-residue insertion, corresponding to an accommodation index of *I*_A_ = −0.5^14^. Because the 3-residue insertion breaks the 7-residue repeat pattern, simultaneous formation of both the αS helices and the DHp coiled coil is not compatible with a straight, continuous helix. Furthermore, since the DHp bundle is highly stable and has its coiled coil always formed, a bistable conformational switch emerges between two conformations—the inactive and the activated structure (Fig. 3B).

In the inactive, unphosphorylated state, the αS/DHp helices are continuous, and the αS helices do not form a coiled coil. The *a*–*d* residues of the αS helix are located on the outside of the helix pair, oriented away from each other. The supercoil of the two αS helices is right-handed, and their N-terminal ends are splayed apart by the Rec domains in the 4-5 arrangement. In contrast, in the activated, phosphorylated state, the coiled coil of the αS helices is fully formed, but the helices no longer continue straight into the DHp bundle (Figs. 1E and 3B). Instead, at the αS/DHp junction, the helices are bent to form a knee-like structure. Residues L149–M152 locally unfold in one subunit and wind around A156 in the other subunit, resulting in an overall ~40° kink of the DHp bundle away from the symmetry axis of the αS coiled coil (Fig. 3C). The *a*–*d* residues of the αS helices are in coiled-coil contact until their N-terminal end, facilitated by the Rec domain 4-5-5 arrangement, which enables close contact of α5 and its continuation, αS. The supercoiling of the αS helices has changed handedness from right to left. Consistently, a bioinformatic analysis of αS helix lengths in Rec-controlled HKs reveals a strong preference for multiples of seven residues, as expected for heptad-repeat coiled coils and a preserved accommodation index (Supplementary Fig. 4B).

To visualize the conformational pathway that LvrB needs to undergo between the inactive and activated states, we have tentatively modeled the transition between them, by creating two structural models that connect the two cryo-EM structures (Fig. 3C, D, and Supplementary Fig. 9). With the assumption that the DHp bundle is not dissociating on the timescale of the transition, the only possibility to connect the two end states is a nearly full rotation of the Rec domains and their connected αS helices around each other. On this trajectory, the X-ray structure would represent an intermediate form, similar to the central models, where the two Rec domains have already separated, and the coiled coil has not yet formed. Of note, during the full transition, the translational movement of the Rec domains changes the supercoiling of the αS helices from right-handed to left-handed, with only a minimal rotational twist. Notably, even though the shift from the 4-5 arrangement to the 4-5-5 arrangement on the isolated Rec dimer at first glance appears to be minute, the two Rec domains must dissociate and translate about each other by almost one complete turn (Fig. 3D).

**Fig. 4:**
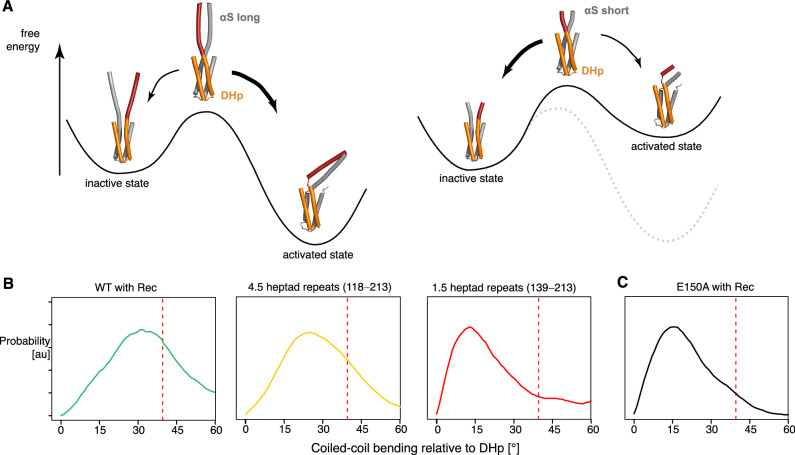
Molecular dynamics probing of the inactive–activated transition of LvrB. **A** Schematic free energy landscape of LvrB DHp/αS. The inactive and the activated states are parts of a bistable switch, separated by a transition state with a straight, continuous coiled coil. Two scenarios are shown, one with a long (left) and one with a short αS helix (right). **B** Effect of the length of the αS helix and the presence of the Rec domain (green) on the bend angle of the coiled-coil domain relative to the DHp domain from MD simulations. In all panels, the red dashed lines show the experimental bending angle observed in the activated cryo-EM structure. **C** Impact of the mutation E150A on the DHp/αS bending angle.

In the next step, we explored the transition between the activated and inactive state and the associated mechanism of knee bending by MD simulations. As the starting point for our simulations, we created a symmetric LvrB conformation where the two αS helices in their coiled-coil conformation are joined as continuous helices to the DHp. To create this model, a single 3_10_-helix turn was inserted at the junction (Q147–N150) to accommodate the 3-residue insertion into the heptad repeats. The resulting model represents a stressed conformation, a transition state between the activated and inactive state (Fig. 4A). This transition state is energetically unfavorable and can relax to either the activated state by knee bending or to the inactive state by dissociation of the αS coiled coil. We also looked for specific structural features in the knee region at the αS/DHp junction and recognized the strictly conserved residue E150, positioned in the “a” heptad position of the αS coiled coil. This residue will likely lead to electrostatic repulsion in the straight conformation, thus adding to the energetic stress and therefore disfavoring the straight conformation.

In full agreement with these expectations, in MD simulations initiated from our stressed transition state model with complete αS helices and the Rec domain, the helix bundle started to bend at the αS/DHp junction, faithfully reproducing the ~40° angle observed in the cryo-EM structure in the ensemble average (Fig. 4B and Supplementary Fig. 10A). A direct analysis of the local secondary structure showed that the local unfolding in the two chains correlates with bending of the knee (Supplementary Fig. 10B, C). The coiled coil was thus stable enough to direct the simulation towards the bent state. Subsequently, we carried out further simulations but with N-terminal truncations to decrease the coiled coil stability. We removed the Rec domain and shortened the length of the coiled coil successively from the N-terminus in alternating steps of 3 and 4 residues, corresponding to a half a heptad repeat (Supplementary Fig. 10A). For helices with more than three heptad repeats present, the knee still showed considerable bending, whereas for helices shorter than three heptad repeats, coiled-coil dissociation occurred instead. We then also tested the role of residue E150. Mutating this residue to alanine substantially reduced bending compared to the WT simulations (Fig. 4C). E150 thus provides an important electrostatic contribution to creating the high-energy transition state, as expected. Clearly, the knee region at the αS/DHp junction, including the conserved E150, is an in-built pivot element to couple αS coiled-coil formation to helix kinking.

### CA domain liberation upon activation

Coiled-coil formation and symmetry breaking have a drastic effect on the CA domains. In the inactive form, the CA domains are held in place via hydrophobic contact with the outward-facing αS-heptad residues. Burying these residues inside the coiled coil upon activation of the protein will thus inevitably result in CA domain dislocation. Consequently, in activated LvrB, one of the domains was found in an altered orientation at the surface of the αS/DHp junction (Supplementary Fig. 5), whereas the other one was not resolved, consistent with it being highly dynamic. The liberated CA domain is supposed to sample a large conformational space, facilitated by its 13-residue DHp-CA linker, and can thus also reach the kinase-active, autophosphorylation-competent conformation. We tentatively modeled the latter by orienting the CA domain relative to the DHp based on reference structure 5LFK^27^. In this conformation, the CA domain is transiently docked onto the DHp bundle by the CA gripper helix, and residue F330 is stacked with F211, and the γ-phosphate of ATP is positioned to phosphorylate H161 (Supplementary Fig. 11A).

**Fig. 5:**
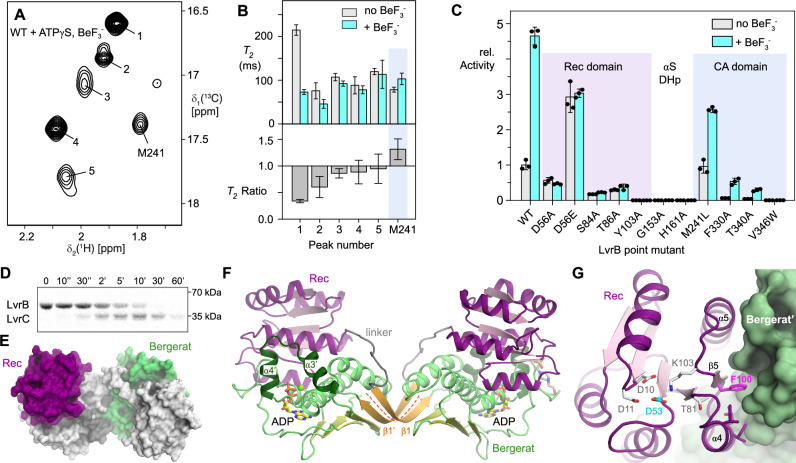
Dynamics and activity of LvrB and structure of the downstream effector LvrC. **A** 2D [^13^C,^1^H]-NMR spectrum of ^13^C-methyl methionine-labeled LvrB in the pseudophosphorylated state. **B** Transverse relaxation times (*T*_2_) of ^13^C-methyl methionine of LvrB:ATPγS, gray bars, and LvrB:ATPγS:BeF_3_^−^, blue bars. The lower panel shows the relative change of *T*_2_(^13^C) upon addition of beryllofluoride. The bar is the result of a non-linear exponential fit to the NMR data. The error bar displays the 95% confidence interval. **C** Activity of LvrB mutants measured by autoradiography, in the absence and presence of beryllofluoride, relative to non-activated WT. All mutant proteins were properly folded (Supplementary Fig. 12). Dots are *N* = 3 experimental data points, bars denote the average, and error bars the standard deviation. **D** Transfer of phosphate from LvrB to LvrC monitored over time by ^32^P-ATP autoradiography. **E** Surface view of the non-phosphorylated LvrC homodimer. One protomer is colored, the other one is gray. **F** Crystal structure of LvrC:ADP. The structure is two-fold symmetric along the β1-β1′ interface (dashed line). **G** Active site and Y-T switch residues of LvrC Rec domain. Residue F100 of the switch and helix α4 interacts with the Bergerat domain of the symmetry-related subunit.

To probe CA domain liberation experimentally, we studied the domain dynamics of LvrB by methyl-NMR spectroscopy. The *T*_*2*_ relaxation times of methionine ^13^C-methyl groups are an effective reporter of local protein dynamics. LvrB has 6 methionine residues, and we assigned M241, the only one within the CA domain, by mutagenesis (Fig. 5A and Supplementary Fig. 11B). The spin relaxation time was then measured for all peaks, both in the absence and in the presence of beryllofluoride (Fig. 5B and Supplementary Fig. 11C, D). Indeed, residue M241 was the only one that increased in *T*_*2*_ upon pseudo-phosphorylation, in agreement with increased flexibility due to domain liberation, while all others decreased.

We then set out to probe our mechanistic model by mutagenesis. Wildtype LvrB features a weak basal activity and a strong activation upon BeF_3_^−^ addition (Fig. 5C and Supplementary Fig. 12). The phosphomimetic mutation D56E rendered LvrB constitutively active, in full agreement with expectations for the phosphorylation-receiving site. The mutation D56A locked the protein at its basal activity. The mutation H161A led to complete inactivity, as expected. For the inverse Y-T switch, our model predicts that mutation of either S84, T86, or Y103 to alanine should prevent full activation. Indeed, the mutation S84A and T86A led to lower basal activity and prevented response to (pseudo)phosphorylation, whereas Y103A resulted in complete inhibition of LvrB activity. Mutation of the pivot residue G153 should prevent the bent active conformation, and indeed, its substitution by the more restrictive alanine resulted in complete inhibition of LvrB autophosphorylation. Finally, the point mutants F330A, V346W, and T340A are expected to destabilize the autophosphorylation-competent state by disrupting π-stacking, preventing CA gripper helix docking, and destabilizing γ-phosphate coordination, respectively. Indeed, each of these mutants displayed a substantially reduced or completely disrupted autophosphorylation efficiency. Altogether, the large set of mutational data fully validates our proposed functional model of LvrB activation.

### LvrB phosphorylates LvrC

To elicit a cellular response, the sensory kinase LvrB must transfer the phosphoryl group from H161 with high specificity to the Rec domain of a downstream RR with gene regulatory function. In order to identify the cognate downstream partner among the 50 RRs in *L. interrogans*^28^, a targeted structural modeling approach was employed to identify candidates with the highest probability to bind LvrB (Supplementary Fig. 13A). In a second step, the correct cognate RR was identified by experimental phosphotransfer profiling (Supplementary Fig. 13B)^29^. Only one protein, encoded by gene *lic11110*, and hereafter termed LvrC, was successfully and rapidly phosphorylated by LvrB (Fig. 5D).

Under pseudophosphorylation conditions in vitro, an LvrB_2_:LvrC_2_ complex formed (Supplementary Fig. 13C); however, multiple attempts to crystallize or cryo-vitrify this transient complex were unsuccessful. Nevertheless, these attempts resulted in crystals of LvrC, allowing us to determine its structure to a resolution of 2.5 Å (Fig. 5E, F and Supplementary Fig. 13D–F, Supplementary Table 2). LvrC possesses an N-terminal Rec domain followed by a domain with a Bergerat fold (Supplementary Fig. 13G). This fold was first described for the *Bacillus subtilis* anti-σ factor RsbW, which reversibly sequesters RNA polymerase σ factors to control gene expression^30^. The homodimeric structure of LvrC shares the Bergerat topology, with the dimer formed by interactions of the β1 strand and α1 helix. LvrC’s Rec domains are canonical and contribute to dimerization in the crystallized form via their 4-5-5 surfaces (Fig. 5G). The intermolecular interaction is mostly hydrophobic (I86, I90, L93, I102), with the Y-T switch residue F100 protruding into a hydrophobic cavity on the other protomer and thus pointing away from the phosphorylation acceptor D53. The high similarity to RsbW and SpoIIAB (Supplementary Fig. 13H)^30,31^, suggests that LvrC acts as a Rec-regulated anti-σ factor by reversibly releasing and sequestering a yet unknown σ factor. Phosphorylation of D53 by LvrB likely causes the release of the Rec via the canonical Y-T switch. This mechanism thus explains the massive transcriptional reprogramming controlled by the Lvr phosphorelay during *Leptospira* infection^7^.

## Discussion

**Fig. 6:**
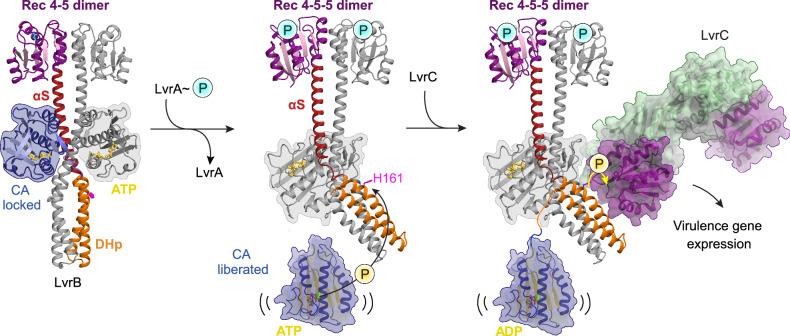
Model of LvrB function. In the unphosphorylated state, LvrB forms a symmetric dimer, with the αS helices splayed apart and the CA domains locked to the DHp core. Upon phosphorylation of residue D56 by upstream LvrA, αS helices form a coiled coil with symmetry breaking at its C-terminal end, resulting in CA domain liberation.The liberated CA domain autophosphorylates LvrB at residue H161, with the phosphoryl group subsequently taken up by the Rec domain of LvrC.

Our work has resolved 3D structures of a full-length histidine kinase in both the inactive and the activated state, uncovering the activation mechanism of this important class of signaling proteins (Fig. 6). The inactive state of LvrB is symmetric, with the two non-phosphorylated Rec domains keeping the αS helices apart. In this configuration, the CA domains are rigidly held onto the central DHp bundle in a geometry that prevents them from auto-phosphorylating residue H161. Phosphorylation of the Rec domains generates a mechanical signal via the inverse Y-T switch, which is an intriguing variation of this allosteric switch. The resulting rearrangement of the Rec domains to form a 4-5-5 dimer, accompanied by a change of the αS helix supercoiling from right- to left-handed, allows for αS coiled-coil formation, concomitant with an asymmetric bending. Coiled-coil formation liberates one of the catalytic CA domains into an activated form, competent to phosphorylate H161. Overall, a mechanical signal is allosterically propagated from the Rec domains to the HK core over a distance of more than 50 Å. Most HKs cycle between kinase- and phosphatase-active states^9^. We propose that LvrB follows this paradigm, with the two activities segregated between conformational states: the asymmetric state being kinase-active, and the symmetric state potentially serving as the phosphatase-active form.

In addition, we have successfully identified the putative anti-σ-factor protein LvrC as the cognate downstream partner, thus establishing the Lvr signaling pathway as a TCS/σ-factor hybrid system. σ/anti-σ partner-switching systems allow bacteria to respond to stress swiftly by triggering global transcriptional reprogramming^32^. The Rec-Bergerat architecture of LvrC is ideally suited to connect a fast-transmitting sensory phosphorelay to a global reprogramming regulator, which may be critical for *Leptospira* to adapt rapidly to the transition from environmental to animal host milieu. To illustrate a plausible architecture of the LvrB–LvrC signaling complex, we tentatively docked our LvrC structure onto activated LvrB. Because the liberated CA domain is not resolved in this structure, this docking was possible without steric clashes in a canonical position. We note that the signaling complex might involve a specific arrangement of the CA domains, inviting future structural characterization. Furthermore, based on the position of the catalytic H161 residues in the activated structure, where the *trans* site is sterically not accessible, LvrB autophosphorylation likely occurs *in cis*, a feature that will also require future experiments to be conclusive.

LvrB can be taken as a paradigmatic basis for the family of Rec-controlled histidine kinases (Rec-HKs). Previously referred to as hybrid RRs^33^, they rather represent a subclass of canonical HKs^9,34^, many of which are animal or plant pathogens^7,18,19,35–37^. Beyond HK classification, the mechanistic insights disclosed in LvrB also contribute to understanding the vast set of related receptors and effectors that harbor αS signaling helices between regulatory and core domains^9,13^. Different mechanisms of αS reorientation have been suggested for HKs, involving piston or scissoring motions^9,11^. In particular, it has been pointed out that the mismatch between the heptad repeats of αS helices and the DHp bundle, with the associated structural weakness at the junction, may be central to creating a bi-stable switch^14^, and we show that this is indeed the case for LvrB. A structure with a kinked DHp bundle has previously been resolved for the artificially designed HK YF1, but the role of the bending was unclear^38^. The activated form of LvrB observed here aligns well with YF1, including the pivot residue G153. With LvrB being a full-length HK where both bent and unbent conformations have been resolved, the mechanistic role that symmetry breaking has in HK activation and signaling has been elucidated.

LvrB exhibits a mechanism by which Rec-phosphorylation-induced coiled-coil formation is coupled to CA domain liberation, the prerequisite for autophosphorylation. Since coiled coils are predicted to be present in the large majority of Rec-HKs (Supplementary Fig. 4C), our results provide the cornerstone for understanding many unexplored cellular processes, and will strongly assist in the development of antibiotics and agrochemicals targeting Rec-HKs. Based on the two-fold symmetric inactive structure of LvrB, we speculate that a symmetric molecule that inserts between the αS helices and forces them apart could effectively inhibit LvrB’s kinase activity. The same principle would be applicable to PhcR, the prototypical Rec-HK in the crop killer *Ralstonia solenacearum* that is crucial in plant infections^37^.

## Methods

### Cloning and protein expression

The codon-optimized sequence of LvrB (*lic11708*) was synthesized and inserted into a pET-28a vector with a Kanamycin resistance cassette by GenScript (Piscataway, NJ, USA). Shorter LvrB constructs and point mutants were generated from the pET-28a-lvrB vector following the Q5 Site-Directed Mutagenesis Kit protocol (E0554, New England Biolabs) using suitable primers and the results verified by sequencing. Primer sequences are provided as Source data. The *E. coli* Top10 strain was used for cloning purposes. Bacteria were allowed to grow in lysogeny broth (LB; 10 g tryptone, 5 g yeast extract, 10 g NaCl) supplemented with 50 μg/ml Kanamycin (LB-Kan) and LB-Kan-containing agar plates at 37 °C. After successful cloning, plasmids were extracted from cells using the ZR plasmid miniprep kit (Zymo Research) and used to transform the expression strains *E. coli* BL21(DE3) or similarly efficient LEMO cells. For protein expression, adequate amounts of LB-Kan media were inoculated with 1% pre-culture of transformed cells. Grown cultures were induced with 0.5 mM isopropyl 1-thio-β-D-galactopyranoside (IPTG) at an OD_600_ of 0.6–0.8. The incubation temperature was reduced to 23 °C for overnight protein expression. Cells were harvested by centrifugation at 8000 × *g* for 10 min at 4 °C. Pellets were stored at −80 °C or lysed immediately. For crystallization purposes, apart from full-length LvrB, an insert encoding the CA domain alone (LvrB_CA_, residues K216-S380) was subcloned into pQE80L (Qiagen), transformed into *E. coli* TOP10F’ cells, grown at 30 °C until OD_600_ ~0.8 and induced overnight with 1 mM IPTG at 20 °C. Cells were harvested and lysed as before. For NMR experiments, *E. coli* LEMO cells were transformed with the pET-28a-lvrB vector and grown in deuterated M9 minimal media (6.8 g/L Na_2_HPO_4_, 3 g/L KH_2_PO_4_, 0.5 g/L NaCl, 1 g/l ^15^NH_4_Cl, 4 g/L glucose, 2 mM MgSO_4_, Solution Q, and Vitamin mix) instead of LB. Furthermore, for the methyl-labeling experiments, L-methionine-(methyl-^13^C) was added 1 h before induction with IPTG.

The codon-optimized sequence of LvrC (*lic11110*) was inserted into a pQE80L vector with an Ampicillin resistance cassette. *E. coli* TOP10F’ strain was used for cloning and protein expression. Cells were allowed to grow in LB supplemented with Ampicillin (LB-Amp) at 37 °C until OD600 of 0.7–0.8. Induction was achieved by the addition of 0.5 mM IPTG, and the temperature was reduced to 23 °C overnight. Cells were harvested by centrifugation at 8000 × *g* for 10 min at 4 °C. Pellets were stored at −80 °C or lysed immediately.

### Protein purification

All purifications of LvrB were performed at 4 °C. Cell pellets were homogenized in lysis buffer containing immobilized metal affinity chromatography (IMAC) loading buffer (400 mM NaCl, 30 mM Tris-HCl, 5 mM MgCl_2_, 20 mM Imidazole, pH 7.5), complemented with 1 mM PMSF, 100 μg/ml lysozyme from chicken egg white, and DNase. Cell lysis was performed with a microfluidizer set at 3 bar. The lysate was ultracentrifuged at 14,000 × *g* for 1 h, to remove cell debris and suspended particles. The clear supernatant was applied to a 5 mL Ni-sepharose column (Cytiva) pre-equilibrated with IMAC loading buffer. Bound protein was eluted with a linear gradient of IMAC elution buffer (400 mM NaCl, 30 mM Tris-HCl, 5 mM MgCl_2_, 500 mM imidazole, pH 7.5) using an ÄKTA Pure system (Cytiva). Fractions containing the desired protein were pooled and concentrated to a volume of 5 mL or below and then loaded on a HiLoad 16 or 26/600 Superdex 200 pg gel filtration column (Cytiva) pre-equilibrated with SEC buffer (200 mM NaCl, 30 mM HEPES, 5 mM MgCl_2_, pH 7.5). The concentrations of the collected samples were quantified by UV absorption with a NanoDrop 2000 spectrophotometer (Thermo Fisher Scientific) and either used freshly or stored at −80 °C.

Purification of LvrC was performed at 4 °C. Cell pellet homogenization and IMAC chromatography were done as for LvrB, but in buffers with a pH of 8.0. Fractions containing the desired protein were pooled and dialyzed at 4 °C overnight in SEC buffer (see above) at pH 8.0 in the presence of 0.02 mg/mL TEV protease. A second IMAC step identical to the first one was performed, and the flow-through was collected. Pooled fractions were concentrated to a volume of 5 mL and then loaded on a HiLoad 26/600 Superdex 200 pg gel filtration column (Cytiva) pre-equilibrated with SEC buffer at pH 8.0. Concentrated samples were either used freshly or stored at −80 °C.

### Pseudo-phosphorylation of the Rec domain with beryllofluoride

Stock solutions of beryllium fluoride were prepared by mixing a 1 M sodium fluoride (NaF) water solution and a 0.1 M beryllium chloride (BeCl_2_) water solution in a 1:1 ratio. The pH was adjusted by the addition of 10 mM Tris-HCl at pH 7.0. The beryllium fluoride solution was added to the target protein at least 15 min prior to the experiments, wherein the modified protein was required.

### Isothermal titration calorimetry (ITC)

Measurements were performed with a MicroCal VP-ITC calorimeter (Malvern Instruments) at 25 °C. Samples were diluted in ITC buffer (100 mM NaCl, 20 mM Tris-HCl, 5 mM MgCl_2_, 1 mM DTT, pH 7.5). A total of 30 injections of 10 μl each (besides the first injection with 2 μl) were made with a spacing time of about 400 s. Experiments were limited by the solubility of LvrB and therefore did not reach ideal c-values. Consequently, the binding stoichiometry was fixed to the 1:1 ratio (2 ATP per LvrB dimer), in order to not to overfit the data. Thermograms were analyzed with Origin v7.0 using a 1:1 binding model.

### Multi-angle light scattering (SEC-MALS)

Samples at the concentration indicated in the text were applied to analytical size exclusion columns equilibrated overnight in SEC buffer (see above) and separated at 25 °C with a flow rate of 0.5 ml/min, with the help of an Agilent 1260 series HPLC system equipped with high performance autosampler and multi-wavelength detector. MALS and differential refractive index (dRI) were measured by a Heleos II 8-angle detector (Wyatt) and an Optilab rEX detector (Wyatt), respectively. The device was calibrated using a 2 mg/ml BSA solution in the same SEC buffer. The data were collected and processed with the ASTRA software (Wyatt).

### Differential scanning fluorimetry (DSF)

Unfolding experiments were carried out by nanoDSF dye-free protocol using a Prometheus NT.48 instrument (NanoTemper Technologies). The samples contained 20 μM LvrB in 50 mM NaCl, 30 mM Tris-HCl at pH 7.5, 5 mM MgCl_2_, and the ligands indicated in the text. The mixes were incubated for 20 min at 20 °C before DSF measurements. Equilibrium equations were fitted to the data with ProFit 7.0 (Quantum Soft).

### Plate-reader fluorimetry

Fluorescence of TNP-ATP (50 nM) in the presence and absence of protein (10 μM) was measured in a Corning 384-well plate format, 25 μL per well, with the aid of a Tecan Spark plate reader (Tecan Life Sciences). Data was acquired at *λ*_em_ = 550 nm with *λ*_ex_ = 408 nm, with a Z-height of 18.8 mm and a sensor gain of 110. A protein-dependent increase in TNP-ATP intensity is indicative of binding^39^.

### Autophosphorylation assays

For SDS-PAGE autoradiography, LvrB was incubated in enzymatic reaction buffer (200 mM NaCl, 20 mM Tris-HCl, 5 mM MgCl_2_, 1 mM DTT, pH 7.5) with 1 mM ATP (Sigma Aldrich) and 5 μCi [γ-^32^P]-ATP (3000 Ci mmol^−1^, Hartmann Analytic) at room temperature. Aliquots were taken at defined timepoints, quenched with SDS-PAGE loading dye, and subsequently loaded on precast 4–20% gradient polyacrylamide gels (BIO-RAD). Wet gels were exposed to a phosphor screen (0.5–3 h) before imaging using a Typhoon FLA 7000 imaging system (GE Healthcare). Background-subtracted SDS-PAGE and autoradiograph band intensities were determined with ImageJ (NIH) and plotted with ProFit 7.0 (Quantum Soft).

For dot-blot autoradiography, reactions were prepared similarly as for the SDS-PAGE procedure, with 10 μM LvrB and 200 μM ATP, in triplicate per condition (with or without 5 mM beryllofluoride). Aliquots were taken after 10 min, quenched with SDS-PAGE loading dye, and pipetted on Amersham Protran 0.2 μm nitrocellulose membrane (Cytiva) laid on Immobilon blotting filter paper (Merck Millipore) to absorb excess liquid. The dots (1.5 μl) were conveniently stained in blue, allowing precise pipetting without using a vacuum chamber. The membrane was allowed to dry for 15 min, and subsequently washed three times for 5 min in 25 mM phosphoric acid, at which point the dots became colorless. Then, the membrane was washed three times for 5 min with distilled water, and dried for 0.5–1 h at room temperature. Dry membranes were exposed to a phosphor screen overnight before imaging using a Typhoon FLA 7000 imaging system (GE Healthcare). The dot intensities were determined with a custom Python script.

### PhosTag acrylamide gel

Reactions were done with 5 µM LvrB in 25 mM Tris-HCl pH 8.5, 250 mM NaCl, 10 mM MgCl_2_, 1 mM ATP. At indicated times, the reaction was stopped by adding SDS-PAGE loading buffer, and separated by Phos-tag acrylamide gel electrophoresis. Phos-tag acrylamide (FUJIFILM Wako Chemicals) gels were prepared according to the manufacturer’s instructions, with minor modifications. Running gels contained 10% (w/v) 29:1 acrylamide:N, N′-methylene-bis-acrylamide, 375 mM Tris pH 8.8, 0.1% (w/v) SDS, copolymerized with 25 μM Phos-tag acrylamide and 50 μM ZnCl_2_. Stacking gels contained 4% (w/v) 29:1 acrylamide:N, N′-methylene-bis-acrylamide, 125 mM Tris pH 6.8, 0.1% (w/v) SDS. Gels were run at 4 °C at 150 V, and stained with Coomassie blue.

### LvrB phosphotransfer profiling

To prioritize the list of cognate response regulators (RRs) potentially phosphorylated by LvrB, all the RRs encoded in *L. interrogans*’s genome were systematically evaluated using an in-silico approach. The 3D structure of the receiver Rec domain of each RR (domains delimited according to Pfam Response_reg model PF00072 / InterPro entry IPR001789) was predicted in complex with LvrB’s DHp domain (residues G153 to G217) with AlphaFold2^40^, using default settings (ColabFold v1.5.5 AlphaFold2 implementation) and a custom-generated paired multiple sequence alignment (MSA). The MSA was the same for all modeling tasks^41^, filtered to include 250 DHp:Rec sequence pairs (each RR/HK pair belonging to the same operon). Models were ranked according to the interface pTM score (ipTM)^42^.

Selected RR Rec domains were cloned into a modified pQE80L plasmid (Qiagen Kit) by RF cloning^43^. Primer sequences are provided as Source data. RRs were ultimately expressed as a fusion to an N-terminal 6xHis-tag, followed by a TEV protease cleavage site. Recombinant proteins were overexpressed in *E. coli* TOP10F’ (Invitrogen) grown in LB and induced overnight at 18 °C with 0.5 mM IPTG (when cultures reached exponential growth rate). After harvesting, bacterial pellets were resuspended in lysis buffer (Tris 50 mM, pH 8, NaCl 0.5 M, imidazole 20 mM, 1 mg/ml lysozyme, and EDTA-free Protease Inhibitor Cocktail (Merck)), and incubated at room temperature for 20 min. For DNA shearing and to complete cell disruption, the extracts were sonicated and centrifuged for 60 min at 17,000 × *g*. The cleared lysates were incubated with 0.5 ml of Ni-NTA agarose beads (Cytiva) for 30 min at 4 °C. Ni-NTA beads were washed three times with buffer A (Tris 50 mM, pH 8, NaCl 0.5 M, imidazole 20 mM). Elution was achieved by loading the Ni-NTA beads into empty PD-10 columns (Cytiva), followed by 2.5 ml Buffer B (Tris 50 mM, pH 8, NaCl 0.5 M, imidazole 500 mM). Eluates were then loaded into PD-10 columns pre-equilibrated with reaction buffer (25 mM Tris, pH 8.0, 125 mM NaCl, 1 mM DTT), and eluted with 3.5 ml of reaction buffer. Desalted proteins were concentrated, quantified by nanodrop, and stored at −80 °C until usage. Purifications were evaluated by SDS-PAGE; if purity was <90%, a final size exclusion chromatography step was performed (Superdex75, Cytiva).

For the determination of the phosphotransfer activity of LvrB toward selected RR Recs^44^, LvrB was incubated with 5 mM BeF_3_^−^ for 15 min at room temperature, prior to the addition of 0.5 mM ATP and 30 µCi of [γ^33^P]-ATP (ARC). After 30 min the protein was desalted with a PD-10 column into the reaction buffer. Phosphotransfer reactions were performed with 5 µM radiolabeled phosphorylated LvrB and 5 µM of each RR species, in reaction buffer at room temperature. Reactions were stopped at the corresponding times by mixing the samples with SDS-PAGE loading buffer. Reaction products were separated in reducing SDS-PAGE, and visualized with a BAS-MS imaging plate (Fujifilm) after 3 h of exposure, in a Typhoon FLA 9500 scanner (GE). For phosphotransfer kinetics from LvrB to LIC11110 and LIC12807, the same procedure described above was followed, stopping the reaction at the indicated times.

### Cryo-EM grid preparation, data acquisition, and processing

For the inactive structure, LvrB was diluted to 2 mg/ml (~44 μM, final concentration) in SEC buffer (see above) and mixed with ATPγS at a 1:1.5 molar ratio. For the active structure, LvrB was diluted to 1.5 mg/ml (~33 μM, final concentration) in SEC buffer and mixed with ADP at a 1:2 molar ratio and 10 mM beryllofluoride. Cryogenic samples were prepared using Vitrobot Mark IV (Thermo Fisher Scientific) at 95% humidity and 4 °C. Three microliters of the samples were applied to Quantifoil R1.2/1.3 200-mesh copper holey carbon grids, previously glow-discharged for 30 s at 20 mA, and blotted for 3–5 s with Whatman filter paper. The grids were flash-frozen in liquid ethane and then stored in liquid nitrogen before clipping and loading into the microscopes. Movies were recorded with SerialEM on a Glacios microscope (Thermo Fisher Scientific) operated at 200 kV and equipped with a Gatan K3 direct electron detector (DED). Images were recorded with a pixel size of 0.878 Å. Each micrograph was dose-fractionated to 42 frames under a dose rate of: 14.5 e^−^/Å^2^ per second, with a total exposure time of 4.00 s, resulting in a total dose of about 57.9 e^−^/Å^2^ (inactive, dataset 1); 10.9 e^−^/Å^2^ per second, with a total exposure time of 4.87 s, resulting in a total dose of about 53.1 e^−^/Å^2^ (inactive, dataset 2); 10.9 e^−^/Å^2^ per second, with a total exposure time of 5.48 s, resulting in a total dose of about 59.8 e^−^/Å^2^ (activated). Data processing was carried out entirely in cryoSPARC^45^. After patch motion and patch CTF estimation, the micrographs were sorted based on CTF fit resolution, relative ice thickness, anisotropy, and motion, resulting in a total of 5508 micrographs for the inactive LvrB (LvrB:ATPγS, the two datasets combined) and 6732 micrographs for the activated LvrB dataset (LvrB:ADP:BeF_3_^−^).

For LvrB:ATPγS, datasets 1 and 2 were combined to increase the number of available particles. Initially, particles were found by blob picking with a circular or circular/elliptical mask of 70–150 Å and extracted with a box size of 256 pixels. After 2D classification, selected classes from both datasets were used for template picking, which returned 4,131,414 particles from the combined 5508 micrographs. Particles were extracted with a box size of 256 pixels. Multiple rounds of 2D classification, ab initio 3D reconstructions (four classes), and heterogeneous refinement were performed to discard bad particles. The selected 133,625 particles were processed first by homogeneous refinement with C2 symmetry, followed by local refinement, also with C2 symmetry and dynamic mask from homogeneous refinement output set at 0.2 threshold and 3–12 Å range. Further improvements in map quality were obtained by a subsequent non-uniform refinement with C2 symmetry, resulting in a reconstructed map with a resolution of 4.24 Å at 0.143 Fourier Shell Correlation (FSC) cutoff, as determined by cryoSPARC. Maps were globally sharpened according to the B-factor estimated by cryoSPARC.

For LvrB:ADP:BeF_3_^−^, a total of 2,062,480 automatically picked particles were processed similarly to the previous dataset, although C2 symmetry was not enforced. Multiple back-and-forth rounds of 2D classification, ab initio reconstruction, and heterogeneous refinement were implemented and returned better results than conventional 3D classification. Selected 130,240 particles were processed by homogeneous refinement, limiting alignment to 6 Å and increasing the SNR factor to 500. Subsequent local refinement, done with a dynamic mask from homogeneous refinement output set at 0.09 threshold, yielded a map with a resolution of 6.79 Å. The thereby generated volume was used in reference-based motion correction, returning 129,432 improved and unique particles. Local CTF refinement and multiple non-uniform refinements with a smoothened volume (0.02 threshold, 2 dilation, 32 soft padding) as a mask, finally resulted in a map with a resolution of 5.92 Å at 0.143 FSC cutoff, determined by cryoSPARC. Maps were globally sharpened according to the B-factor estimated by cryoSPARC.

### Cryo-EM model building

For the LvrB:ATPγS model, the individual domains of full-length LvrB (pdb IDs 8VC9, 9QL9, 9QJG; solved by X-ray crystallography in this study) were fit into the cryo-EM map. Interactive model building was then performed with Coot and ChimeraX^46,47^, using intra- and inter-chain distance restraints and low Geman-McClure alpha parameters for real space refinement. In several parts of the map, local resolution was sufficiently high to unambiguously confirm amino acid side chain positions. Ligands ATPγS, Mg^2+^, and Mg^2+^-coordinated waters were visible in the map. The Mg^2+^ coordination spheres were restrained with an octahedral geometry. The model was iteratively refined in real space using Phenix^48^, and in reciprocal space using Servalcat^49^. For both approaches, distance restraints were used, defining secondary structure elements and starting models as self-restraints. Validation of the model and model-to-map fitting was performed in Phenix.

Model building for LvrB:ADP:BeF_3_^−^ followed a similar strategy as for the LvrB:ATPγS model. For the coiled-coil αS helices, a model generated by CCBuilder 2.0 was used. Placement and restraining of ADP-Mg^2+^-H_2_Os were aided by the high-resolution structure 3A0T. Only side chains reliably visible in the cryo-EM map were included in the final model, monitoring both the maximization of model-to-map correlation coefficients and the minimization of estimated overfitting.

### LvrB crystallization and structure determination

For the LvrB:AMPPCP:BeF_3_^−^ structure, screening of crystallogenesis conditions was done with JCSG-plus screen (Molecular Dimensions) in sitting-drop 96-well microplates (Greiner) with a nanoliter dispenser robot (Honeybee963, Digilab) at 20 °C. Manual optimization of hits was done in 24-well plates using the hanging-drop vapor-diffusion method. Rod-like crystals of LvrB_CA_ were obtained in 50 mM Tris, pH 8.5, 100 mM MgCl_2_, and 15% PEG 4000. Crystals of full-length LvrB were grown in 50 mM Tris-HCl, pH 8.0, 1.5 M ammonium sulfate. Diffraction data were collected on beamline PROXIMA-1 at synchrotron SOLEIL (St Aubin, France) and processed with XDS^50^ and Aimless^51^. The crystal structure of LvrB_CA_ was solved by molecular replacement (MR) using an ensemble of models (PDB entries: 1ID0, 1R62, 2C2A, 3A0Y, 4CGZ, and 4Q20) with Phaser^52^. This model was instrumental in solving the structure of full-length LvrB by MR Phaser, which found six copies in the asymmetric unit (ASU). Using this partial solution, six copies of a homology model of the Rec domain (PDB 1MT5 served as template) were subsequently positioned in the ASU. A final model of the full-length LvrB structure was obtained through iterative cycles of model building with Coot^53^ and refinement with phenix.refine^54^, during which the central helical region of the protein (αS helix and DHp domain) was unambiguously defined in the Fourier difference electron density maps.

For the LvrB_CA_ and LvrB_CA_:ADP structures, purified LvrB_CA_ was concentrated to 18 mg/ml and crystallized using the sitting-drop vapor diffusion method. Sets of 3-drop MRC plates were prepared with a Gryphon robot (Art Robbins Instruments). LvrB_CA_ was first crystallized at a drop concentration of 9 mg/ml in the presence of ATPγS at a 1:1.5 molar ratio in the mother liquor containing 0.2 M Ammonium sulfate, 0.1 M Bis-Tris at pH 6.5, 25% w/v PEG 3350. Surprisingly, the resulting structure contained ADP, probably due to spontaneous hydrolysis, and no Mg^2+^. LvrB_CA_ was then crystallized again at a concentration of 6 mg/ml in the presence of AMPPCP at a 1:1.5 molar ratio in mother liquor containing 0.1 M MMT buffer (Malic acid, MES, and Tris) at pH 7.0, 20% w/v PEG 1500. The resulting structure did not contain any ligand. Soaking of the crystals with ATPγS for 2 h at room temperature did not yield holo structures. X-ray diffraction data were collected at the Swiss Light Source (SLS), Villigen, Switzerland, and processed with XDS^50^ and Aimless^51^. The crystal structures of LvrB_CA_:ADP and apo LvrB_CA_ were solved by molecular replacement (Supplementary Fig. 14), using the AlphaFold2 model of LvrB_CA_ extracted from the full-length structure prediction. For both crystal structures, phases and models were further improved by manual model building using Coot^53^ and refinement in REFMAC5^55^ and phenix.refine^54^.

### Bioinformatics

Coiled-coil propensities were predicted with one or more of the available algorithms, DeepCoil, LearnCoil, MarCoil, and PCoils^56–58^, as indicated in the text. To establish histograms of the Rec-DHp linker length distribution in Rec-DHp proteins, sequences belonging to proteins with a Rec-DHp module in their architecture were obtained by querying the UniProt and InterPro databases^59,60^. For each sequence, the Rec-DHp linker length was measured starting from the conserved KP residues in the Rec domains and ending at the conserved H box in the DHp domain. To establish histograms of the orientation of the Y/F residues, an exhaustive set of Rec domain proteins belonging to the Pfam PF00072 family was obtained by querying the InterPro database^60^. For each corresponding entry, the associated PDB files were downloaded. Using a custom Python script relying on the BioPython library^61^, the resolved amino acid sequences were extracted directly from each atomic model. Non-canonical amino acids, including post-translationally modified residues, were converted to their canonical equivalents (e.g., BFD and PHD were converted to Asp) prior to exporting the sequences in FASTA format. All extracted sequences were aligned against the PF00072 hidden Markov model (HMM) seed using HMMER’s hmmscan tool^62^, enabling automated and high-precision identification of the conserved D and Y residues. The hmmscan output was subsequently parsed using another Python script to map the identified D and Y residues back onto the corresponding PDB structures. For structures in which both residues were successfully identified, the script (i) calculated the distance between the Cα atom of the D residue and the last atom of the Y residue (which could be either a Tyr or a Phe), and (ii) classified each structure as representing either a non-phosphorylated or (pseudo)phosphorylated Rec domain, thereby enabling subgrouping of the dataset. The same script was used to generate the histograms shown in the Supplementary Material.

### Molecular dynamics simulation

As a starting model for the MD simulations of coiled-coil bending relative to the DHp domain, a symmetric state of LvrB was generated by joining the DHp domain of the crystal structure with the coiled-coil helix αS of activated LvrB. Since there is a mismatch between the αS and the DHp heptad repeats (3-residue insertion), residues Q147 and N150 were connected by a 3_10_ interaction. The model was subsequently refined with REFMAC5. Individual simulations were performed for (i) seven variants with residues R118–F213, E122–F213, D125–F213, R129–F213, A132–F213, E136–F213, and L139–F213, (ii) full αS helices including the Rec domains (residues K3–F213), (iii) same as ii with mutation E150A in both chains. Furthermore, simulations were performed for individual Rec domains, comprising residues K3–R129, originating either from our activated crystal structure or the inactive cryo-EM structure. The tilt angle was measured between the principal axes of DHp (C^α^ atoms 139–148) and αS (C^α^ atoms 180–212).

In all systems, missing atoms were added using Modeller10.5^63^, hydrogens and termini were added by the GROMACS tool pdb2gmx. All residues were in their native charge state at pH 7, the nonnative termini were neutral, and the phosphorylated aspartic acid carried a charge of −2. The parameters for phosphorylated aspartic acid were generated by CHARMM-GUI^64^, and the phosphate group was manually modeled to D56 in PyMOL.

All-atom simulations were performed using GROMACS 2023^65^ with the CHARMM36m force field^66^ and TIP4p^67^ water. CHARMM36m was developed to better capture both folded and intrinsically disordered polypeptide segments and is thus a broadly utilized all-atom force field also used to study the effect of mutations and posttranslational modifications on protein structure and function^68,69^. As a control, one simulation batch was repeated in CHARMM TIP3p water model^70^, revealing comparable results.

For each simulation system, 100 steps of steepest descent energy minimization in vacuum were performed, followed by the generation of a dodecahedron box with a minimum distance of 1 nm around the protein, filled with 100 mM NaCl and 10 mM MgCl_2_. Three regimes of energy minimization using the steepest descent algorithm followed. In the first, 500 minimization steps were performed with the whole protein frozen; then only the backbone of the protein was frozen for 1000 steps; and finally, 1000 steps of energy minimization of all atoms were performed. After generation of velocities, the system was equilibrated for 10 ns using position restraints on all protein heavy atoms, followed by 100 ns with position restraints on the protein backbone. In all simulations, a time step of 2 fs was used, and the bonds to hydrogen atoms were constrained using LINCS^71^. The van der Waals interactions were switched to zero over the distance from 0.8 to 1.2 nm, and particle mesh Ewald^72^ was utilized to calculate electrostatics for atoms spaced at least 1.2 nm. The Verlet cutoff scheme^73^ with buffer tolerance of 0.005 kJ/mol/ps per atom was used and the neighbor list was updated every 10 steps. In the position restraint simulations, the Berendsen thermostat^74^ with time constant of 0.5 ps was used to control the temperature at 310 K, and the Berendsen barostat^74^ with time constant of 5 ps and compressibility of 4.5 × 10^−5 ^bar^−1^ kept the average pressure at 1 bar. Thereby, the center of mass of reference coordinates was scaled. In production run simulations, the center of mass of the whole system was linearly removed every 500 steps, the temperature of 310 K was controlled by the Nosé-Hoover thermostat^75^ with a time constant of 0.5 ps, and the pressure of 1 bar was assured by the c-rescale barostat^76^ with the time constant of 5 ps and compressibility of 4.5 × 10^−5 ^bar^−1^. All simulations were performed in three independent replicas initiated from different initial configurations generated by independent pre-equilibration. The coiled-coil models under stress were simulated for 500 ns, while the individual Rec domains were simulated for 5 µs, to capture the movements of Y103/Y87 in- and outwards of the Rec domain. RMSD plots of all simulations are reported in Supplementary Figs. 15 and 16.

### NMR spectroscopy of LvrB

2D [^15^N,^1^H]-TROSY^77^ and 2D [^13^C,^1^H]-HMQC spectra were recorded on Bruker Avance III 700 and Avance III HD 600 MHz spectrometers, equipped with cryogenically cooled 5 mm triple-resonance probes. NMR experiments were recorded at 300–350 μM protein concentration in NMR buffer (200 mM NaCl, 30 mM HEPES, 5 mM MgCl_2_, and pH 7.5) at 293 K. ^13^C *T*_*2*_ transverse relaxation times of ^13^C-methyl methionines were measured using an isotopically labeled sample. Spectra were recorded with different *T* delays (3, 9, 16, 25, 38, 50, 69, 82, 94 ms), and an interscan delay of 1.5 s. Spectra were recorded and processed with TopSpin 3.7. Peak intensities were fitted to an equation of the form *I* = *A* × exp(−*T*/*T*_2_) using ccpNMR v2^78^. The error (95% confidence interval) for *T*_2_ was derived from the fit.

### LvrC crystallization and structure determination

In an attempt to crystallize the LvrB:LvrC complex, LvrB and LvrC were mixed at a 1:2 ratio, at final concentrations of 150 and 300 μM, respectively, in the presence of 1 mM ATPγS and 5 mM beryllofluoride. Then, the mix was centrifuged for 5 min at 16,000 × *g* and filtered through a 0.22 μm membrane. Sets of 3-drop MRC plates for the sitting-drop vapor diffusion method were prepared with a Gryphon robot (Art Robbins Instruments) and incubated at 20 °C. Crystals appeared within a few days in mother liquor consisting of 0.2 M sodium acetate trihydrate, 0.1 M sodium HEPES, pH 7.5, 25% w/v PEG 3350 (SG01-F2, Molecular Dimensions). Crystals were fished after 12 days of growth and flash-frozen in liquid nitrogen. X-ray diffraction data were collected at the beamline PXI of the SLS (Paul Scherrer Institute, Villigen, Switzerland). Data were indexed, integrated, scaled, and merged using XDS^50^ and the CCP4i2 suite^79^. Analysis revealed that the crystals contained LvrC:ADP:Mg^2+^ rather than the intended LvrB:LvrC complex. The crystal structure of LvrC was solved by molecular replacement with Phaser^52^, using a dimeric model of LvrC predicted with AlphaFold2 as a search probe. Phases and atomic coordinates were further improved by multiple cycles of refinement using REFMAC5^55^ and phenix.refine, iterated with manual rebuilding using Coot. Crystal structures were analyzed with PyMOL (Schrödinger, LLC).

### Reporting summary

Further information on research design is available in the Nature Portfolio Reporting Summary linked to this article.

## Supplementary information

Supplementary Information

Reporting Summary

Transparent Peer Review file

## Source data

Source data

## Data Availability

The six structures generated in this work have been deposited in the PDB database under accession codes 8VC9, 9QIH, 9QJG, 9QL9, 9QQW, and 9QR2, as detailed in Supplementary Tables 1 and 2. The following previously published structures from the PDB database have been used: 1ID0, 1MT5, 1R62, 2C2A, 3A0Y, 4CGZ, and 4Q20. The EM data have been submitted to the EM-BD with accession codes EMD-53315 and EMD-53316. The MD data are available at 10.18419/DARUS-5664. Source data are provided as a Source data file. Source data are provided with this paper.

## Code availability

The two custom-made Python scripts used for data analysis are available at 10.18419/DARUS-5664.
