# Supplementary material for: Activation mechanism of the full-length histidine kinase LvrB from pathogenic Leptospira
Source: Nat Commun. 2026 Apr 16;17:5298. doi: 10.1038/s41467-026-71783-4 (PMC13269768; doi:10.1038/s41467-026-71783-4)
Supplement: Supplementary file 1 — Supplementary Information [file 41467_2026_71783_MOESM1_ESM.pdf]

Supplementary Information

Activation mechanism of the full-length histidine kinase LvrB from pathogenic *Leptospira*

Elia Agustoni†, Ariel Mechaly†, Joaquín Dalla Rizza, David Beriashvili, Kristyna Pluhackova, Polina Isaikina, Felipe Trajtenberg, Thomas Müntener, Elsio A. Wunder Jr., Albert I. Ko, Tilman Schirmer, Alejandro Buschiazzo*, Sebastian Hiller*

This file contains:

- Supplementary Figures 1–16
- Supplementary Tables 1–2

Fully uncropped scans of all gel and blot images are provided in the Source Data file.

Supplementary Figures

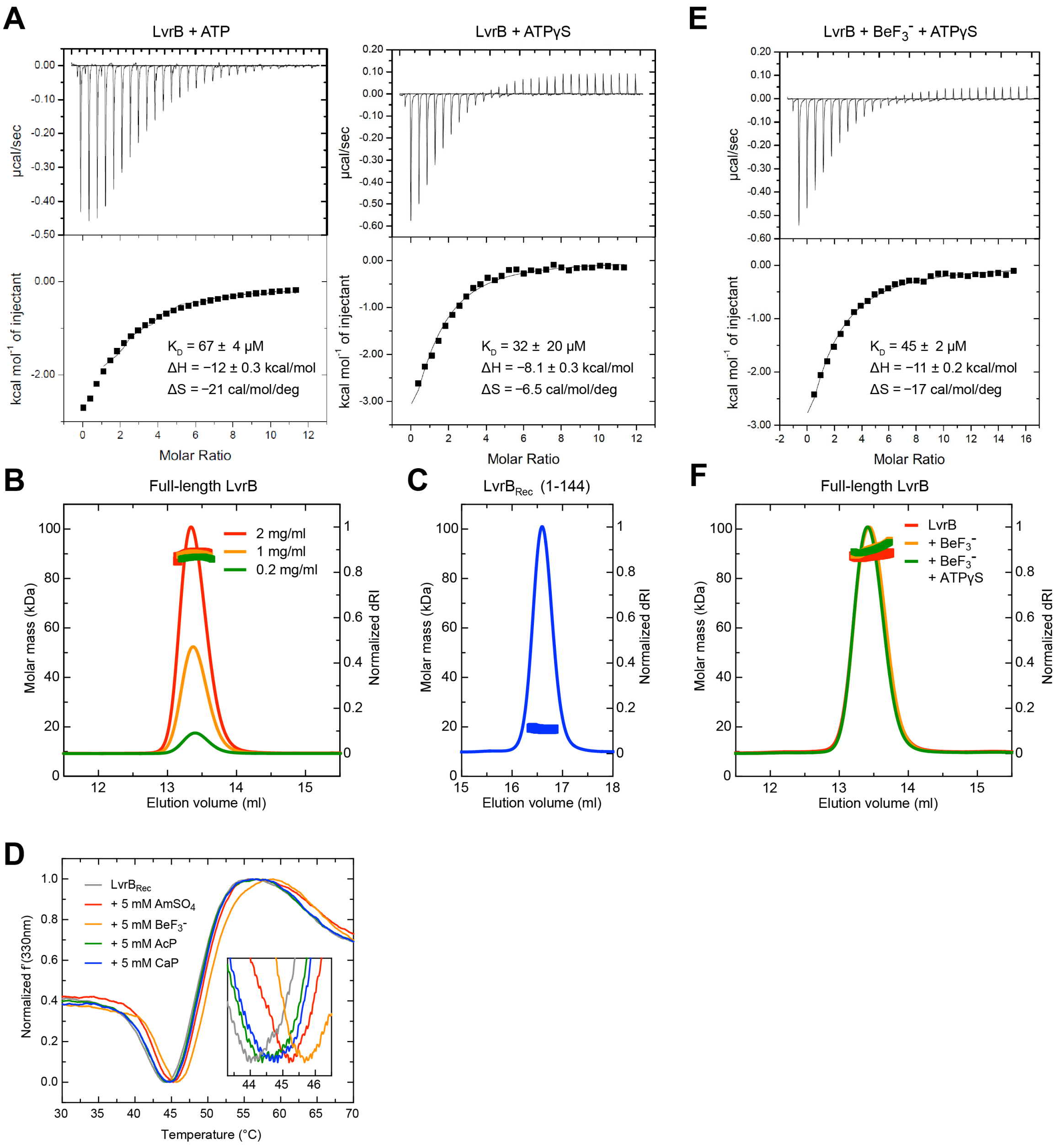

Supplementary Figure 1. Biophysical characterization of LvrB. (A) Binding curves of ATP (left) and ATPγS (right) to 20 µM LvrB obtained by isothermal titration calorimetry (ITC) and fitted with a one-site binding model. The binding stoichiometry was fixed to *N* = 1, based on the cryo-EM and crystal structures of LvrB. (B) SEC-MALS elution profiles of LvrB at different concentrations. LvrB behaves as a homodimer, with no detectable monomeric species even at low concentrations. (C) SEC-MALS elution profile of the isolated Rec domain of LvrB ($LvrB_{Rec}$) at 2 mg/ml concentration, showing that it does not form a homodimer. (D) Melting temperatures (T_m)

of $LvrB_{Rec}$ in absence or presence of the indicated compounds, measured by differential scanning fluorimetry (DSF). (E) Binding curve of ATPγS to 20 μM LvrB preincubated with 5 mM beryllofluoride. Obtained by ITC and fitted with a one-site binding model, *N* = 1. (F) SEC-MALS elution profiles of LvrB at 2 mg/ml concentration, with the indicated ligands. Note that full-length LvrB remains fully dimeric in all conditions tested.

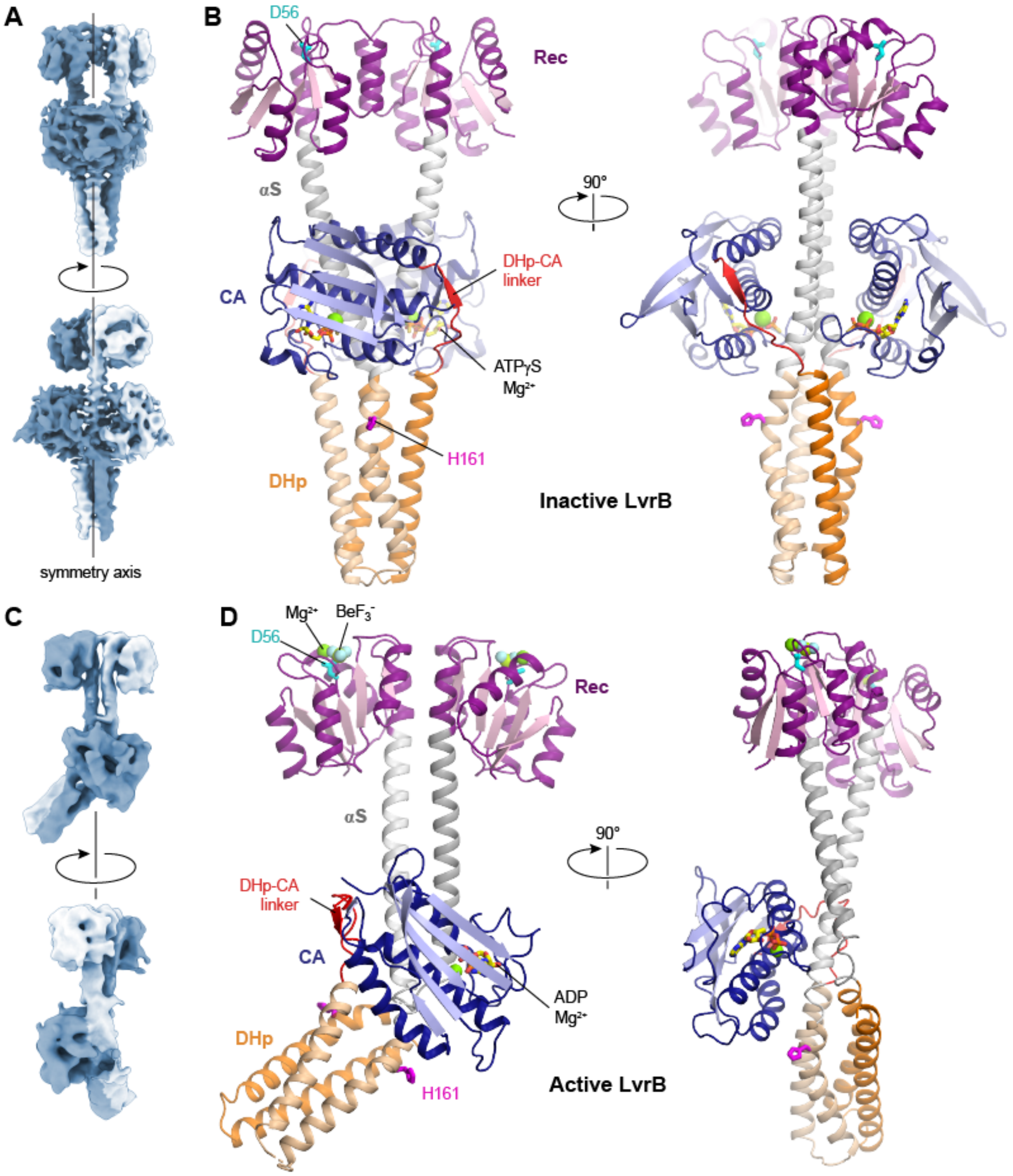

Supplementary Figure 2. Cryo-EM structures of LvrB. (A) Sharpened cryo-EM map of LvrB homodimer in solution at 4.2 Å. (B) Atomic model of LvrB in complex with ATPγS refined within the map. The phosphorylatable residues D56 and H161 are depicted as sticks and colored in cyan and magenta, respectively. (C) Sharpened cryo-EM map of (pseudo)phosphorylated LvrB homodimer in solution at 5.9 Å. (D) Atomic model of LvrB:ADP:BeF_3^- refined within the map. One

CA domain is seen packed against the central helical bundle, the other one did not yield coherent density.

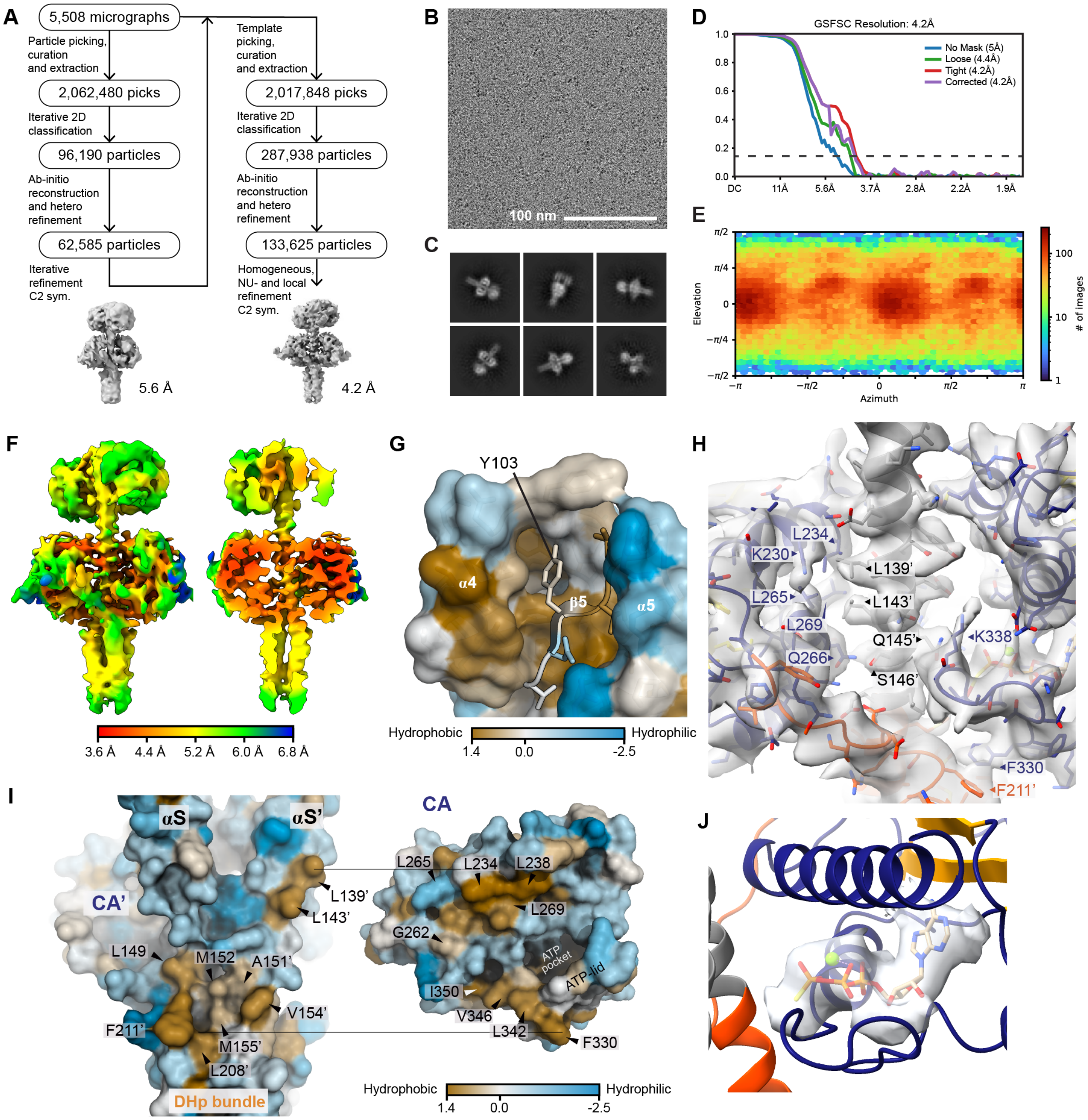

Supplementary Figure 3. Cryo-EM of the inactive state of LvrB. (A) Steps of data processing. (B) Micrograph displaying the sample quality and image contrast. (C) Selected 2D class averages depicting LvrB. The αS helices, Rec, DHp and CA domains are easily recognized. (D) Fourier Shell Correlation (FSC) plot of the dataset, indicating an overall 4.2 Å resolution according to the 0.143 criterion ***(1)***. Blue: unmasked, green: loose mask, red: tight mask, violet: final reconstruction, yielding the indicated gold standard FSC resolution. (E) Viewing angle distribution plot for the final 3D reconstruction. (F) Cryo-EM map of LvrB colored according to the local resolution. The highest resolution is achieved at the center of mass of LvrB. (G) Surface

representation of the α4-β5-α5 surface of the Rec domain, showing β5 in cartoon/stick representation, and highlighting the hydrophobic cavity in which Y103 resides. (H) Detailed view of the residues mediating the interactions between CA domains and the central αS/DHp helical bundle. (I) Surface view of the CA domain–aS/DHp interaction interface, colored according to hydrophobicity. CA domain (right) and αS/DHp helical bundle (left) that interact with each other are displayed next to each other. (J) Coulomb potential density surrounding ATPγS and magnesium.

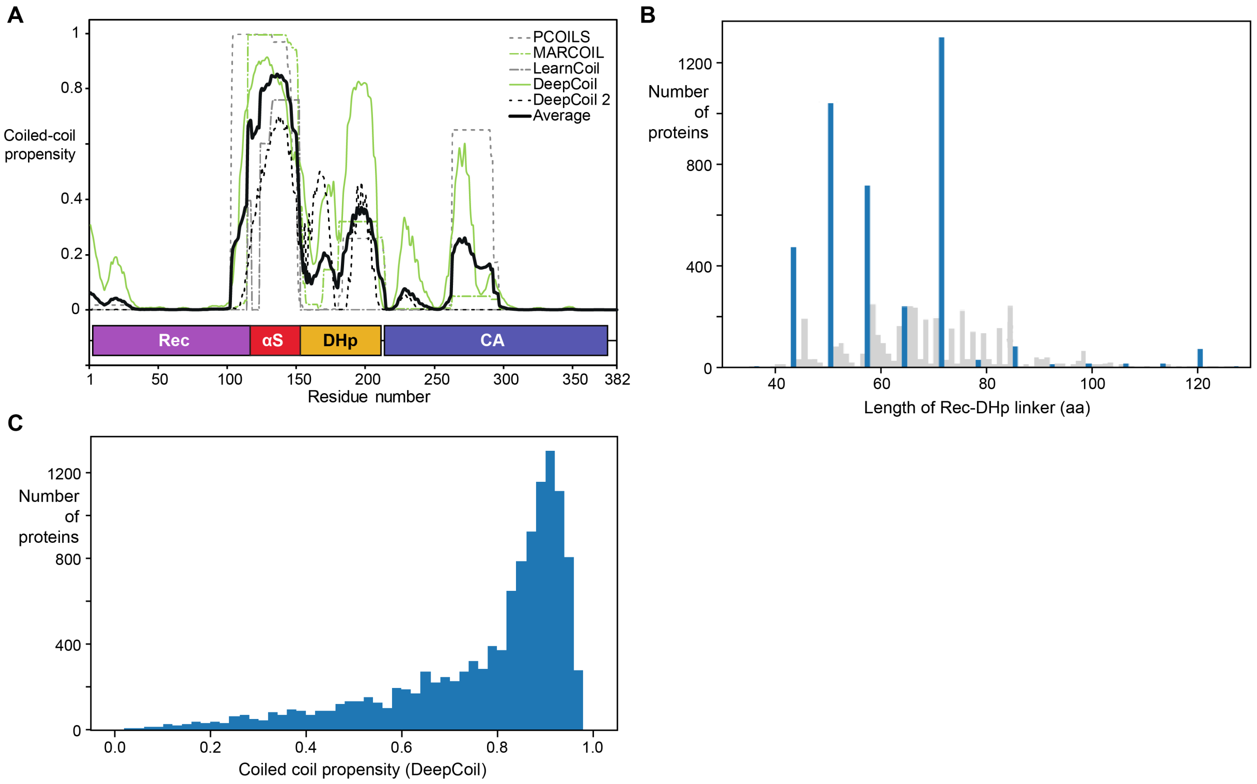

Supplementary Figure 4. Coiled-coil propensity of LvrB and the family of Rec-HKs. (A) Coiled-coil propensity of LvrB calculated with five independent algorithms, as indicated. The average is shown as a black solid line. (B) Histogram of the length of Rec-DHp distances in available sequences. Selected bins spaced by seven residues are highlighted in blue. (C) Distribution of the coiled-coil propensity by the αS helices of prototypical Rec-controlled HKs in the UniProt database (updated 2020).

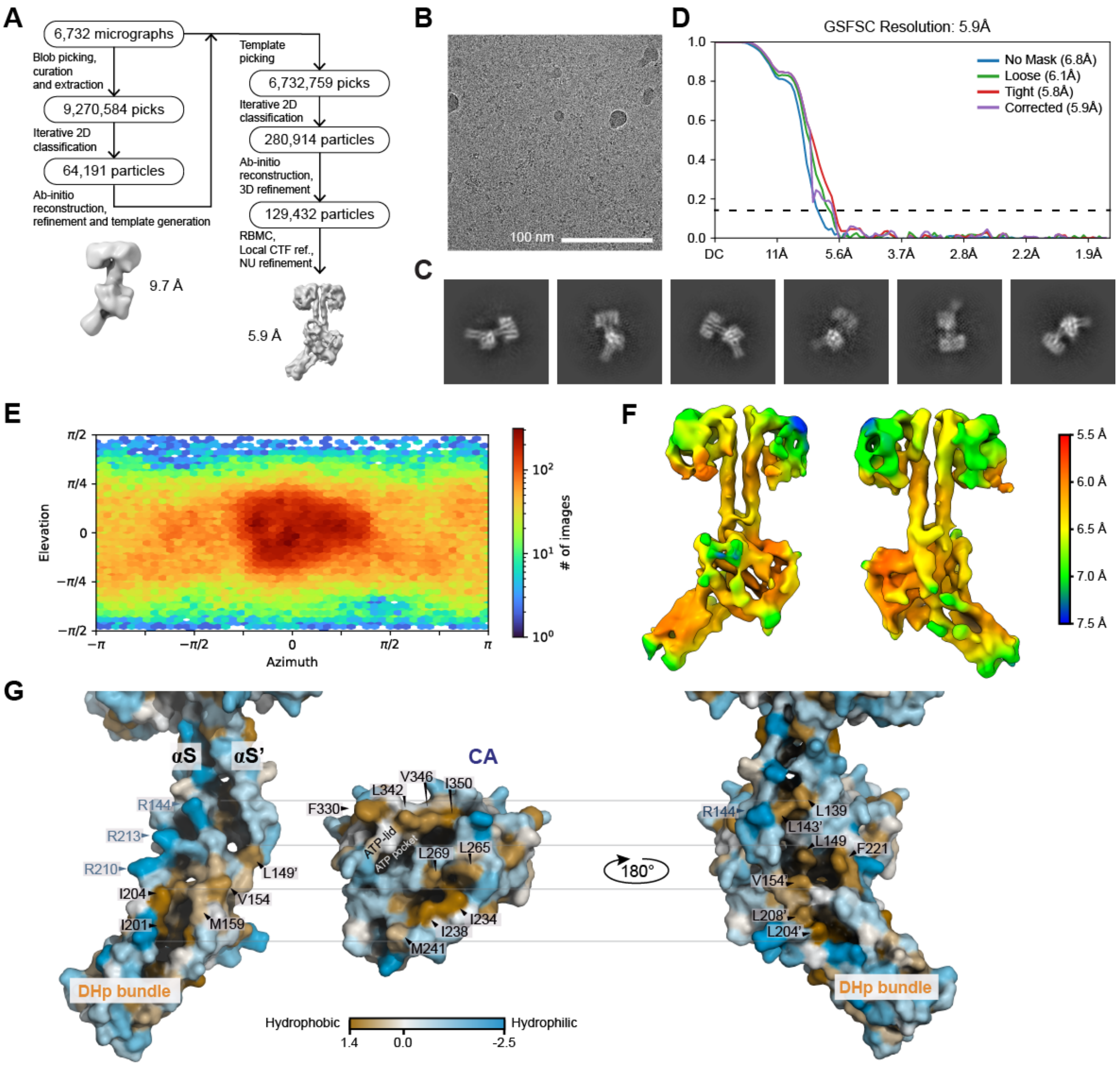

Supplementary Figure 5. Cryo-EM of the active state of LvrB. (A) Steps of data processing. (B) Micrograph displaying the sample quality and image contrast. Particles are distributed homogeneously on the grid, with only few aggregates and ice artifacts. (C) Selected 2D class averages depicting LvrB in its kinase-active form. (D) FSC plots of the dataset, indicating an overall 5.9 Å resolution according to the 0.143 criterion. Blue: no mask; Green: loose mask; Red: tight mask; Violet: final reconstruction, yielding the indicated gold standard FSC resolution. (E) Viewing angle distribution plot for the final 3D reconstruction. (F) Cryo-EM map of LvrB colored according to the local resolution. (G) Surface view of the dimeric aS/DHp helical bundle/CA domain of LvrB colored according to residue hydrophobicity.

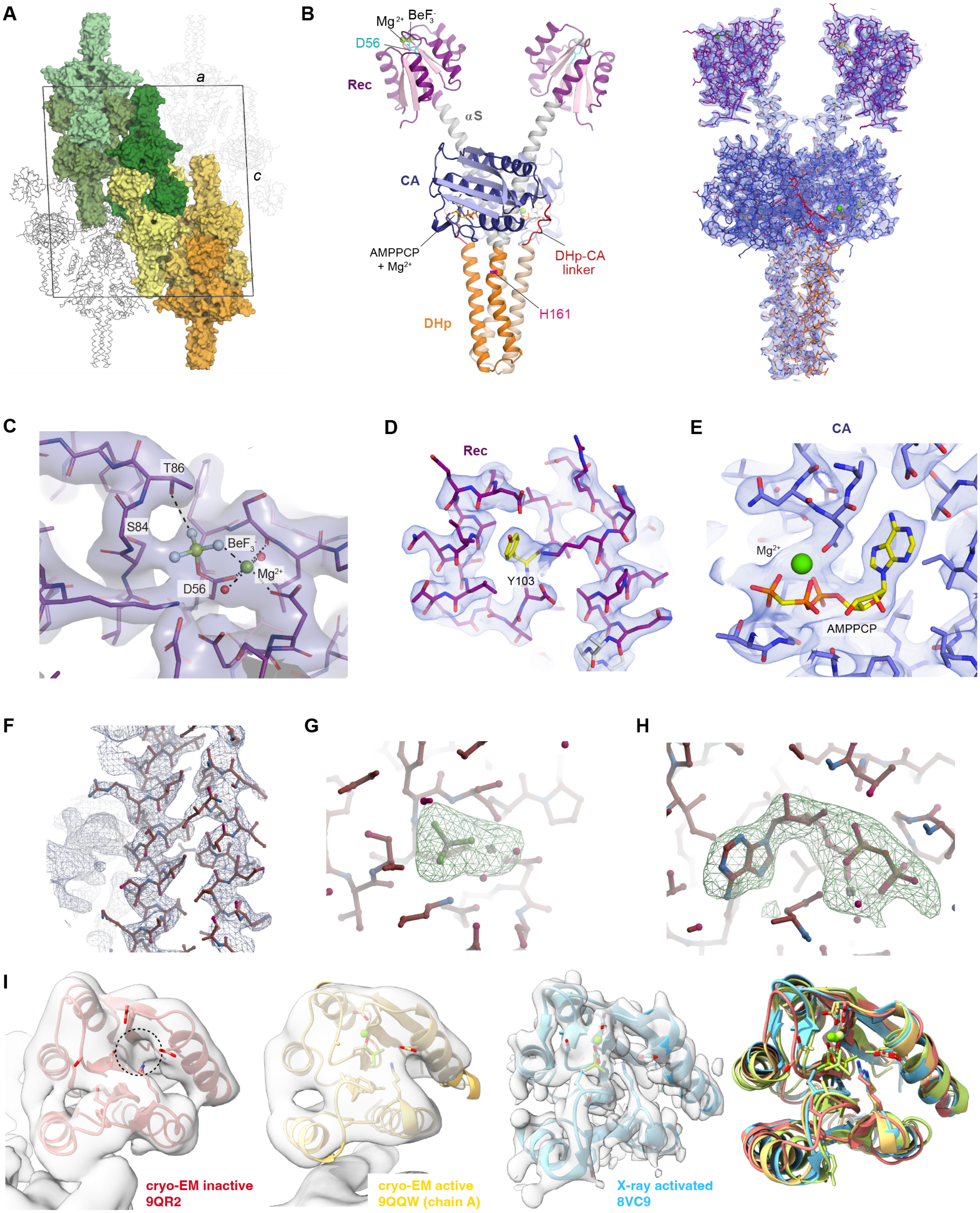

Supplementary Figure 6. Crystal structure of LvrB. (A) Crystal packing of LvrB:AMP-PCP:BeF_3^-, seen along the b axis. Each asymmetric unit contains 6 monomers. The crystals grew

in space-group C2, with a packing formed by intercalation of two LvrB dimers, wherein each Rec domain establishes a large interface with the two CA domains of a neighboring LvrB dimer, and by interaction of Rec and DHp domains between such crystallographic tetramers. (B) Cartoon representation of one LvrB:AMP-PCP:BeF_3^- dimer from the crystal in **A**. Right: $2F_{obs}$-DF_{calc} Fourier electron density map contoured at 1.0σ with the superimposed LvrB dimer. (C) Electron density surrounding the pseudophosphorylated site. Some key residues are labeled. (D) Electron density surrounding Y103 in the Rec domain. (E) Electron density around AMP-PCP:Mg^{2+} in the CA domain. (F) $2F_{obs}$-DF_{calc} electron density map contoured at 1.0σ. (G) Omit map of ligands BeF_3^- :Mg^{2+}, contoured at 3.5σ. (H) Omit map of ligands AMP-PCP:Mg^{2+}, contoured at 3.5σ. (I) Cryo-EM and X-ray densities, along with the corresponding structures of LvrB Rec domains (residues 1–127). Note the empty cavity (dashed circle) near the phosphorylatable residue D56 in the cryo-EM inactive state, occupied by BeF_3^- and Mg^{2+} in the activated conformation. The right-hand panel shows a superimposition of the structures, with additional chain B of 9QQW (green).

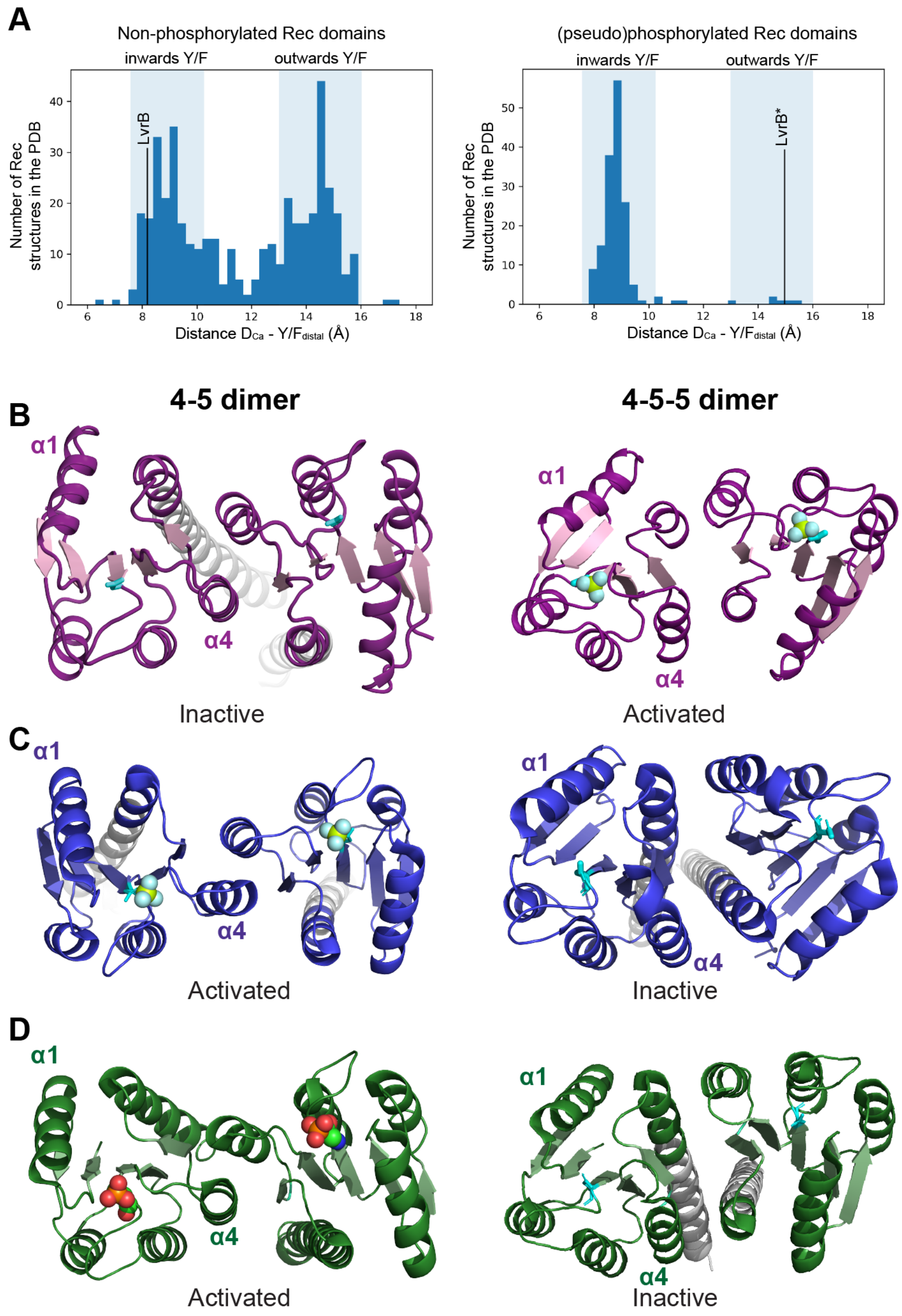

Supplementary Figure 7. Rec domain dimer arrangements. (A) Histograms of the orientation of the Y/F residues in Y-T switches among all Rec structures deposited in the PDB. Unphosphorylated Rec domains display both inward- and outward-facing Y/F residues, whereas (pseudo)phosphorylated Rec domains display almost exclusively inward-oriented Y/F residues. The inverse Y-T switch in LvrB is thus an exception to the rule. (B-D) Rec dimer structures of LvrB (B), DctD (C) and NtrC (D) in the unphosphorylated (left) and phosphorylated states (right) (PDB codes 1L5Y, 1L5Z, 1ZY2, 1NY5). DctD and NtrC have canonical Y-T switches, whereas LvrB has an inverse Y-T switch.

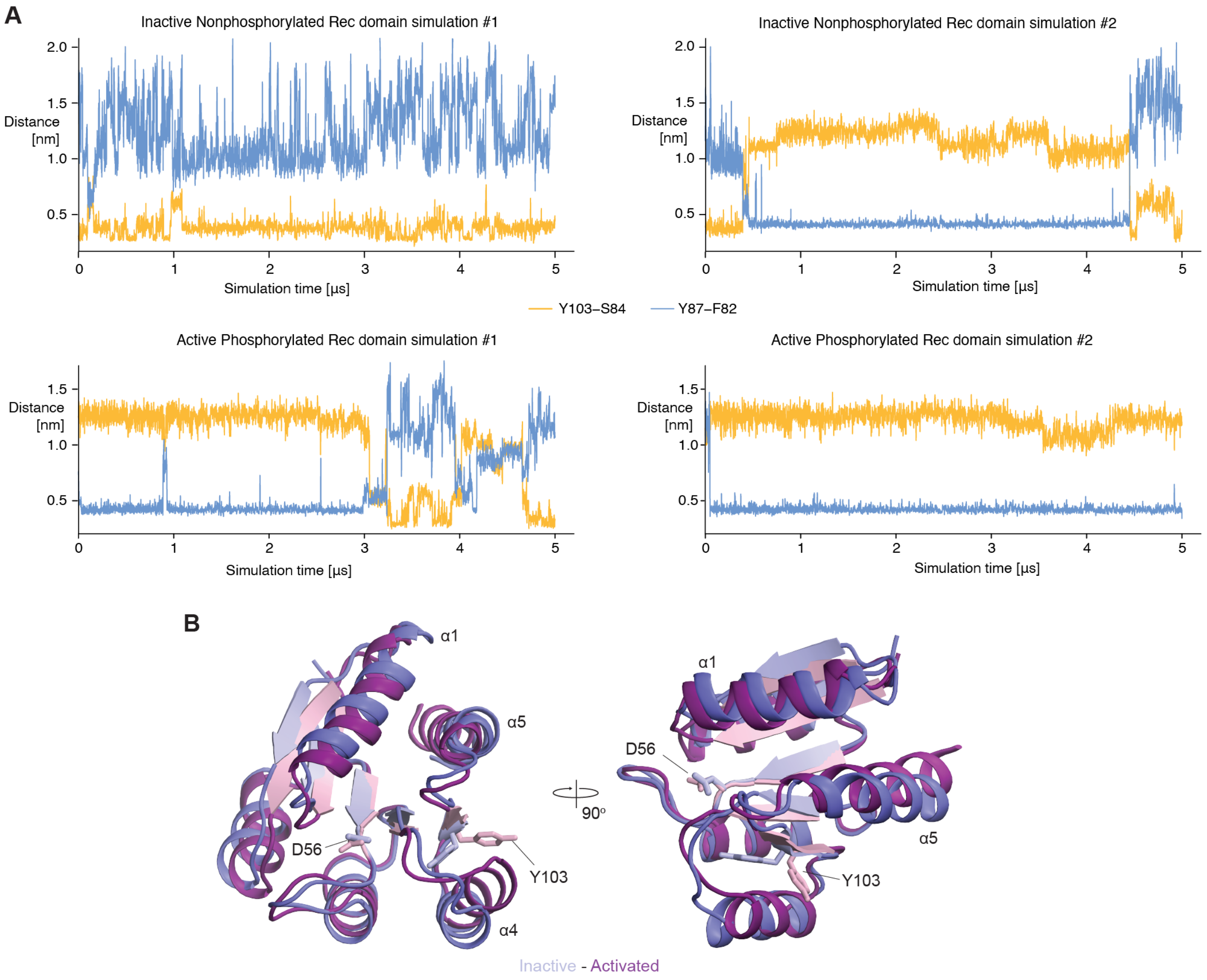

Supplementary Figure 8. Rearrangement of Rec domain structure upon phosphorylation. (A) MD simulations of Y87/Y103 exchange within an isolated Rec domain. The simulations were initiated either from Rec in the inactive/nonphosphorylated state (top row, Y103 "inward", Y87 "outward") or from active/phosphorylated state (bottom row, Y103 "outward", Y87 "inward"). The position of Y103 and S87 is assessed by two characteristic distances. For Y103, the distance between Y103 hydroxyl oxygen and S84 hydroxyl oxygen was monitored, whereas for Y87, the measured distance was the one between its hydroxyl oxygen and the C^{α} atom of F82. (B) Overlay of the inactive (non-phosphorylated) and activated ((pseudo)phosphorylated) structures of the LvrB Rec domain. The side chain conformation of Y103 appears to be coupled to the orientation of helix α4.

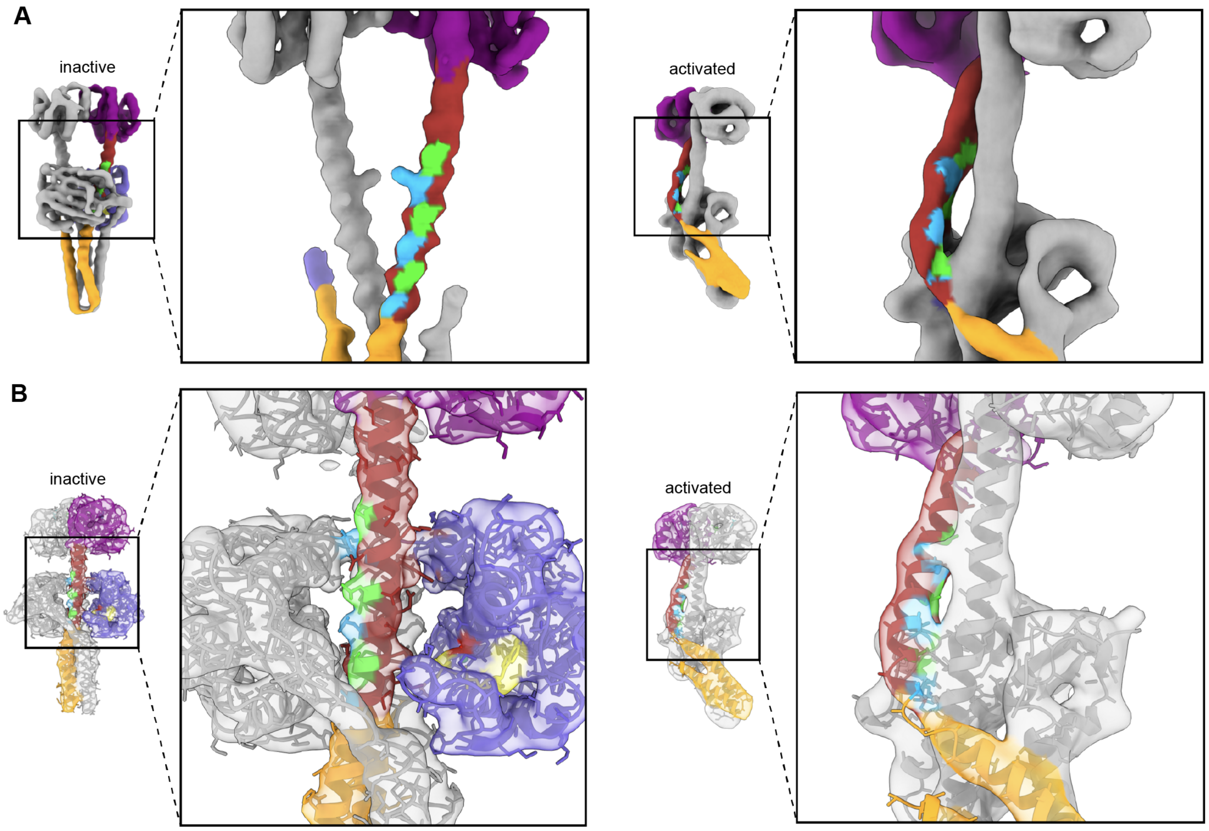

Supplementary Figure 9. Coiled coil formation by the αS helices. (A) Cryo-EM maps colored according to the corresponding models shown in Fig. 1. Left and right panels correspond to the inactive and activated LvrB states, respectively. (B) Same as (A), but with the atomic model shown superimposed on semi-transparent cryo-EM densities and from slightly different viewing angles.

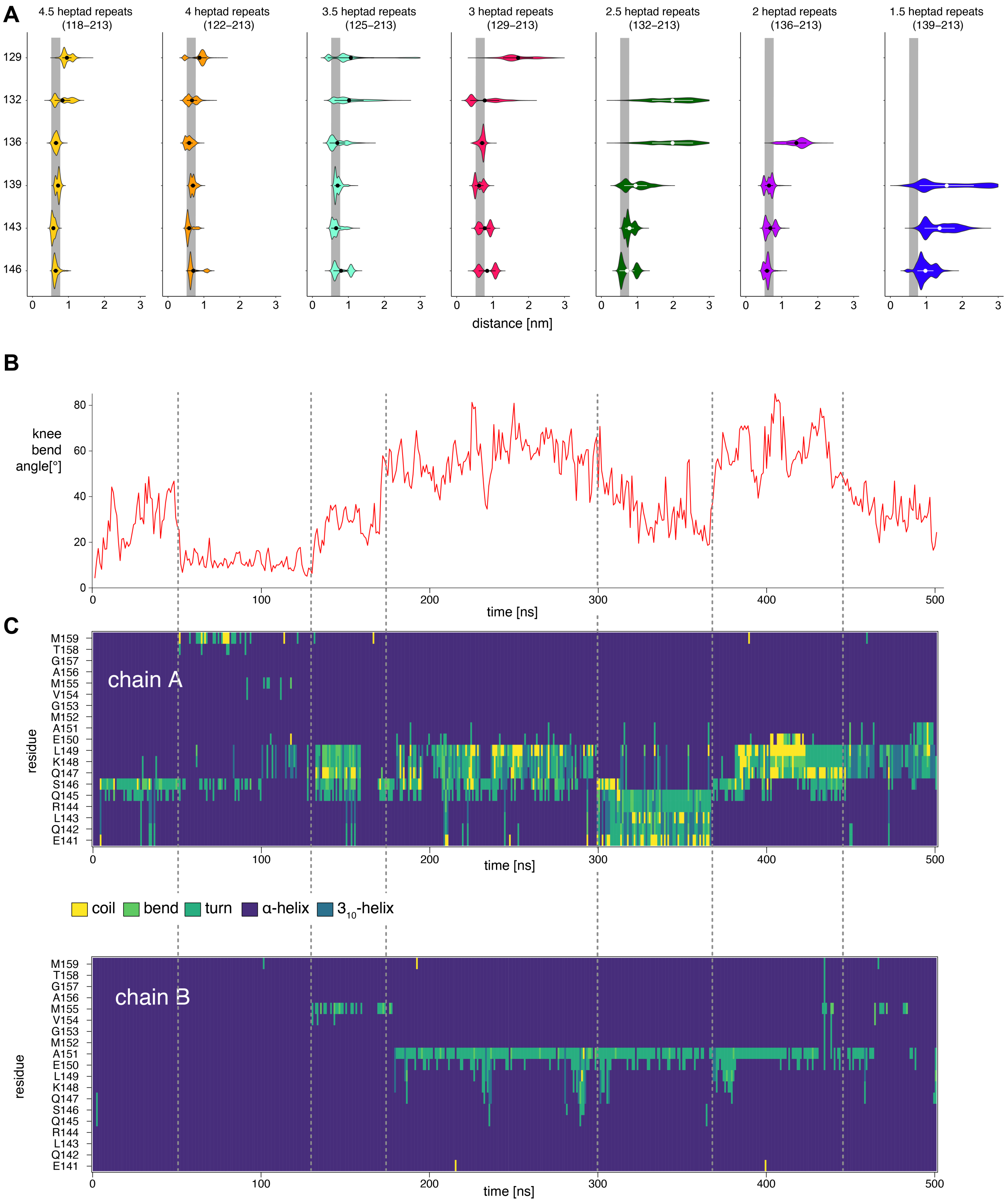

Supplementary Figure 10. Molecular dynamics (MD) simulations of LvrB kinking. (A) C^{α}–C^{α} distance of coiled-coil contacts in models under tension with αS helices of different length. The grey bar corresponds to the canonical distance. Three independent replica MD simulations of 500 ns production run each were performed and data collected in between 250 and 500 ns. For model

129–213, only two replica were evaluated (Supplementary Fig. 16). (B) Correlation between knee bending and local unfolding obtained in a simulation of a model under tension with Rec domains. The time course of the tilt of the coiled coil domain relative to the DHp domain is shown. (C) Secondary structure propensity of chain A and chain B residues 141–159, respectively. The vertical dashed lines crossing through the three panels serve as a time guide. They mark structural transitions that occurred along the trajectory. Five secondary structure types are distinguished according to the backbone dihedral angles that are typical for these elements.

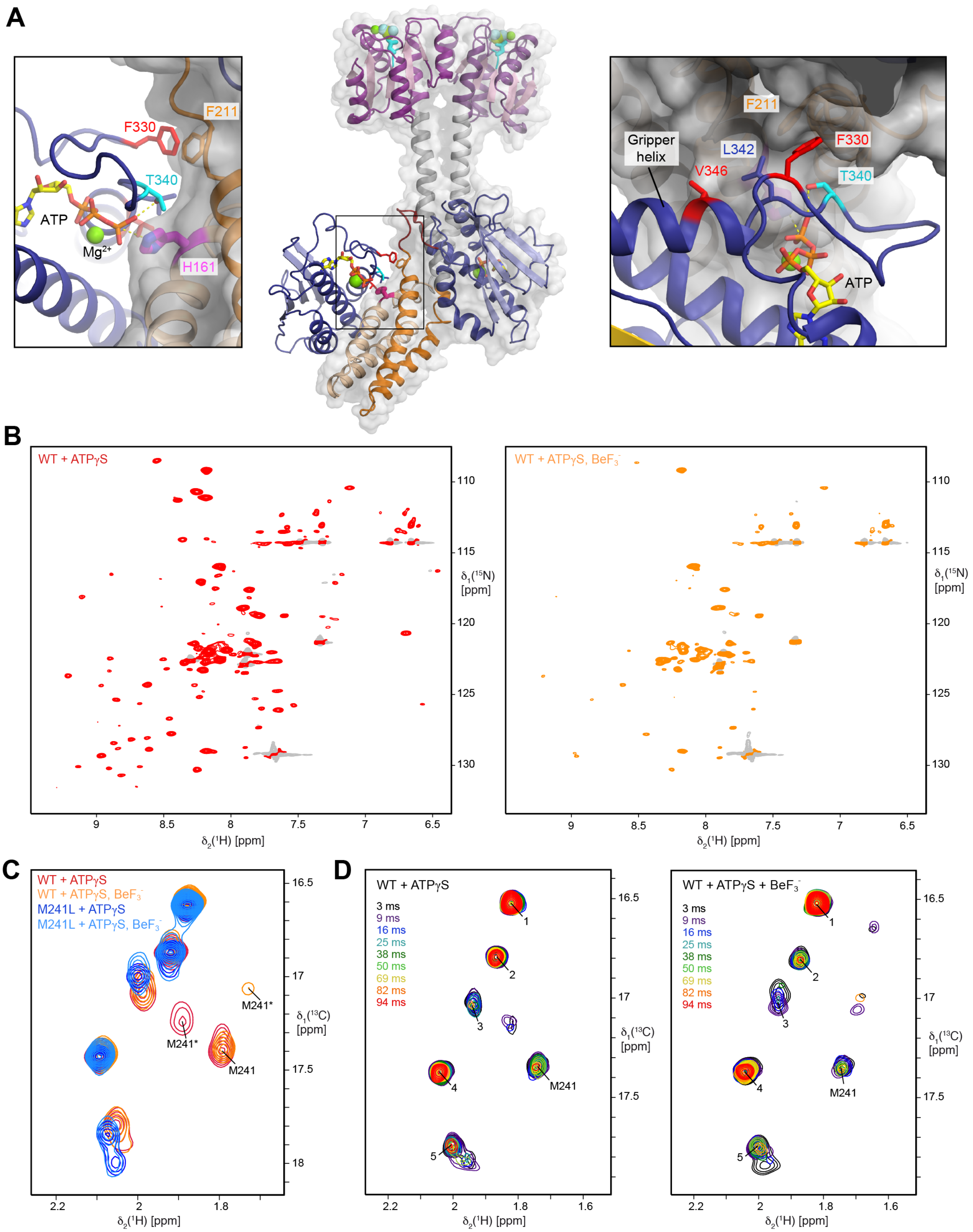

Supplementary Figure 11. Structure and dynamics of the liberated CA domain. (A) Structural model of LvrB in the autophosphorylation-competent form. Residues F330, T340 and V346 on

the gripper helix are involved in CA binding. (B) 2D [^{15}N,^{1}H]-TROSY NMR spectra of 330 µM [*U*-^{15}N]-labeled LvrB in presence of ATPγS (red, left) and of LvrB/ATPγS with beryllofluoride (orange, right). Addition of BeF_3^- causes a pronounced decrease in overall peak intensity, in agreement with loss of the C2 symmetry in the active state. Negative contours are shown in grey. (C) 2D [^{13}C,^{1}H]-HMQC spectra of [^{13}C-Met/^{1}H]-labeled LvrB and [^{13}C-Met/^{1}H]-labeled $LvrB_{M241L}$ in different ligand-bound states, as indicated. The position of the major peak assigned to M241 is not perturbed, while a second, minor peak (asterisk) is shifted upon addition of beryllofluoride. (D) Overlaid series of 2D [^{13}C,^{1}H]-HMQC spectra of [^{13}C-Met/^{1}H]-labeled LvrB, in presence of the indicated ligands, acquired at different relaxation delays. The T_2 relaxation times calculated from these series are shown in Fig. 5B.

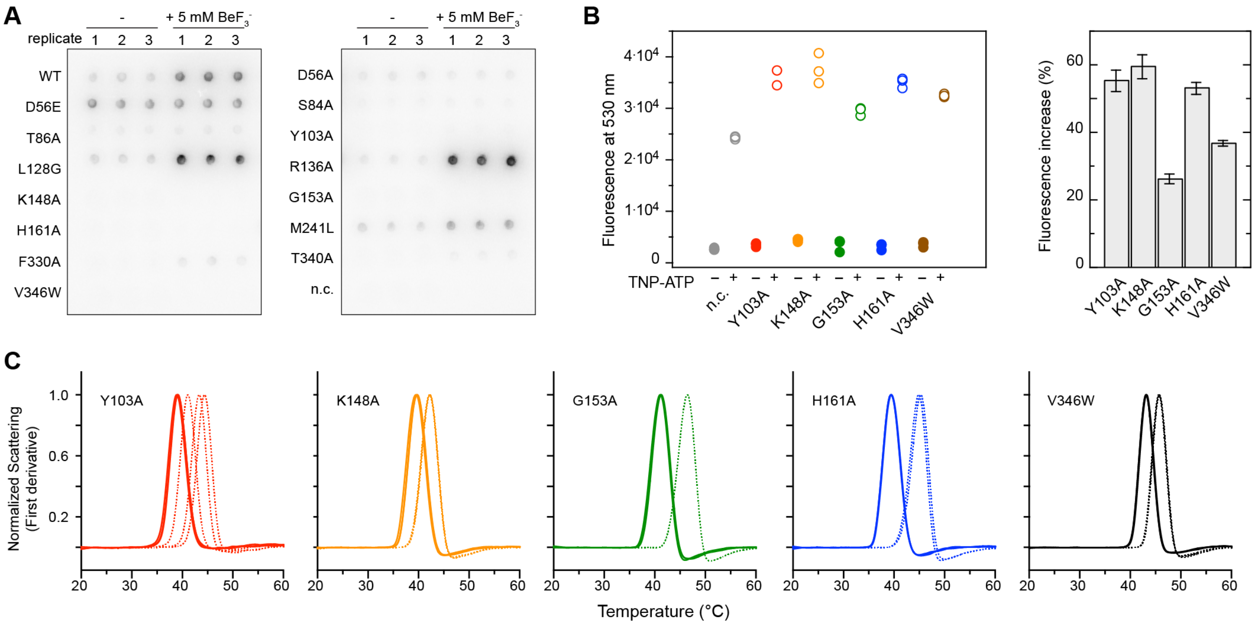

Supplementary Figure 12. Mutational analysis of LvrB function. (A) Dot-blot autoradiographs displaying autophosphorylation of the indicated LvrB constructs after 10 min incubation with ^{32}P-ATP, in triplicate (*n*=3), in absence and presence of beryllofluoride. Integration of these data are shown in Fig. 5C. (B) Fluorescence signal at λ_{em} = 530 nm of mixtures of the indicated LvrB variants, in absence (-) and presence (+) of TNP-ATP. Negative control = buffer. After subtraction of baseline and free TNP-ATP signal, fluorescence of the probe increases significantly upon addition of protein, proving protein-ligand binding. The individual data points for *n*=3 replicates are shown in the lefthand panel. The average and standard deviation of their ratios are shown in the righthand panel. (C) NanoDSF thermograms of the indicated LvrB constructs. The scattering peak values represent the melting temperature (T_m) of the protein in absence (solid curve) and presence (dotted curve) of ATPγS. The marked increase in T_m upon addition of ligand indicates binding of the latter to the protein.

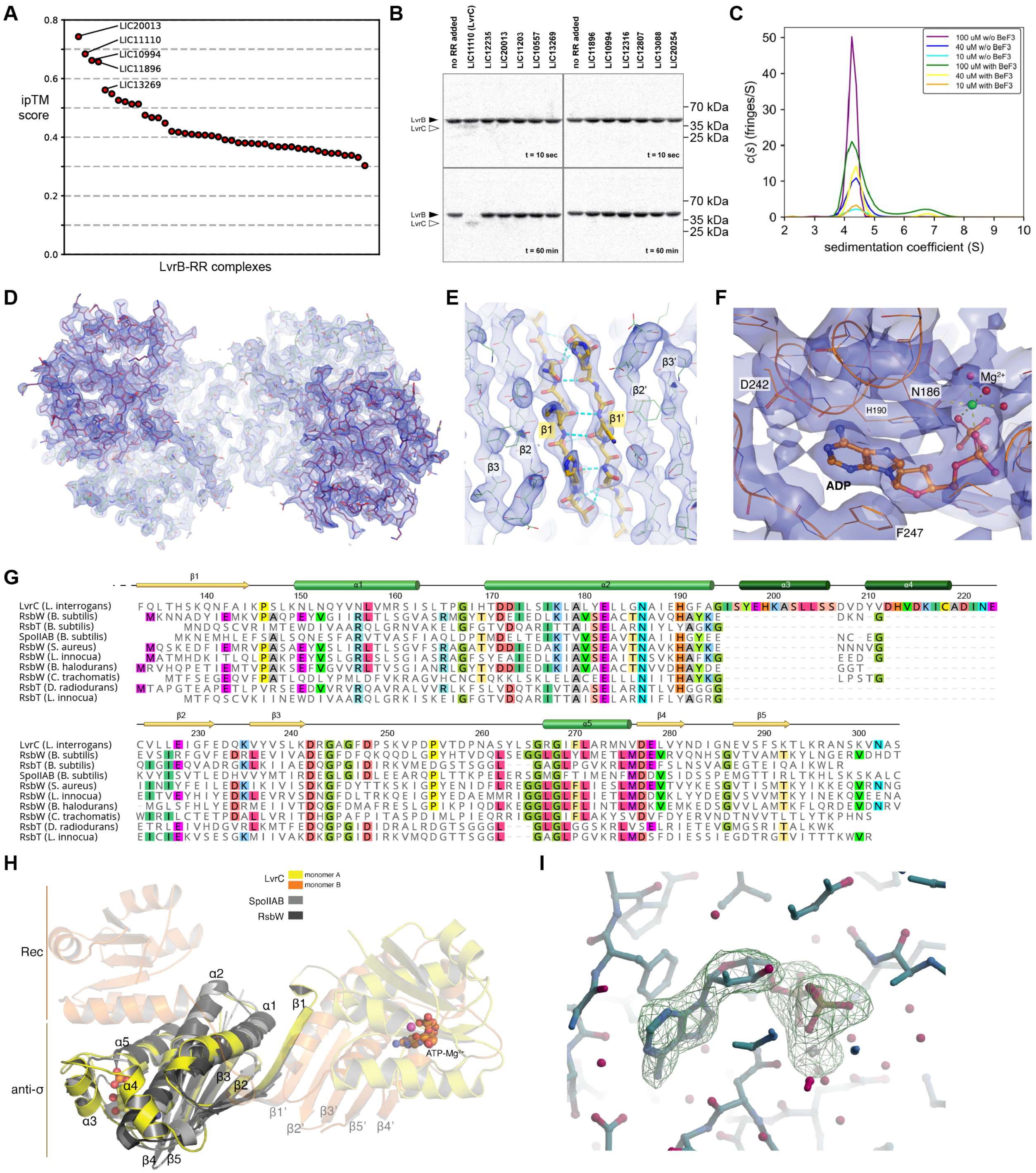

Supplementary Figure 13. LvrC is a putative anti-σ factor protein. (A) Ranked list of interface predicted template modelling (ipTM) scores for 3D structure predictions of LvrB in complex with RRs from *L. interrogans*. The top 5 hits are indicated (B) Autoradiography-based phosphotransfer profiling experiment. ^{32}P-autophosphorylated LvrB is mixed with the indicated proteins. LIC11110 (LvrC) is the only response regulator to accept ^{32}P from LvrB. The experiment was performed n=1

times. (C) SV-AUC plot of LvrB and LvrC at the indicated equimolar concentrations, in absence and presence of beryllofluoride. A subpopulation of LvrB:LvrC complex is observed at higher protein concentrations and only in presence of the phosphomimic, indicating the requirement of the asymmetric conformation of LvrB for LvrC to accept phosphate. (D) Electron density contoured at 1σ surrounding dimeric LvrC:ADP and Mg^{2+}. The Rec domain of each monomer interacts with the RsbW-like domain (RLD) of the other monomer. The dimer is also strongly reinforced by the antiparallel β-sheet edge-to-edge association between β1 strands. (E) Highlight of the antiparallel β-sheet formed by the two interacting LvrC monomers. (F) Fourier density map ($2F_{obs}$-DF_{calc}) surrounding the nucleotide binding site, contoured at 1σ. The octahedral Mg^{2+} coordination sphere is depicted. (G) Sequence similarity analysis between LvrC and known anti-σ factor proteins, such as RsbW, RsbT and SpoIIAB. LvrC differs from the known anti-σ factors by two additional helices, α3 and α4 (dark green), and by the presence of the Rec domain. (H) LvrC dimer superimposed onto anti-σ factors RbsW and SpoIIAB from *B. subtilis*. Secondary structure elements as marked in panel G are indicated. Note that α4 is only present in LvrC, whereas α3 is reduced to a short 3_{10} helix in SpoIIAB and absent in RsbW. (I) Omit map of ligands ADP:Mg^{2+}, contoured at 3.5σ.

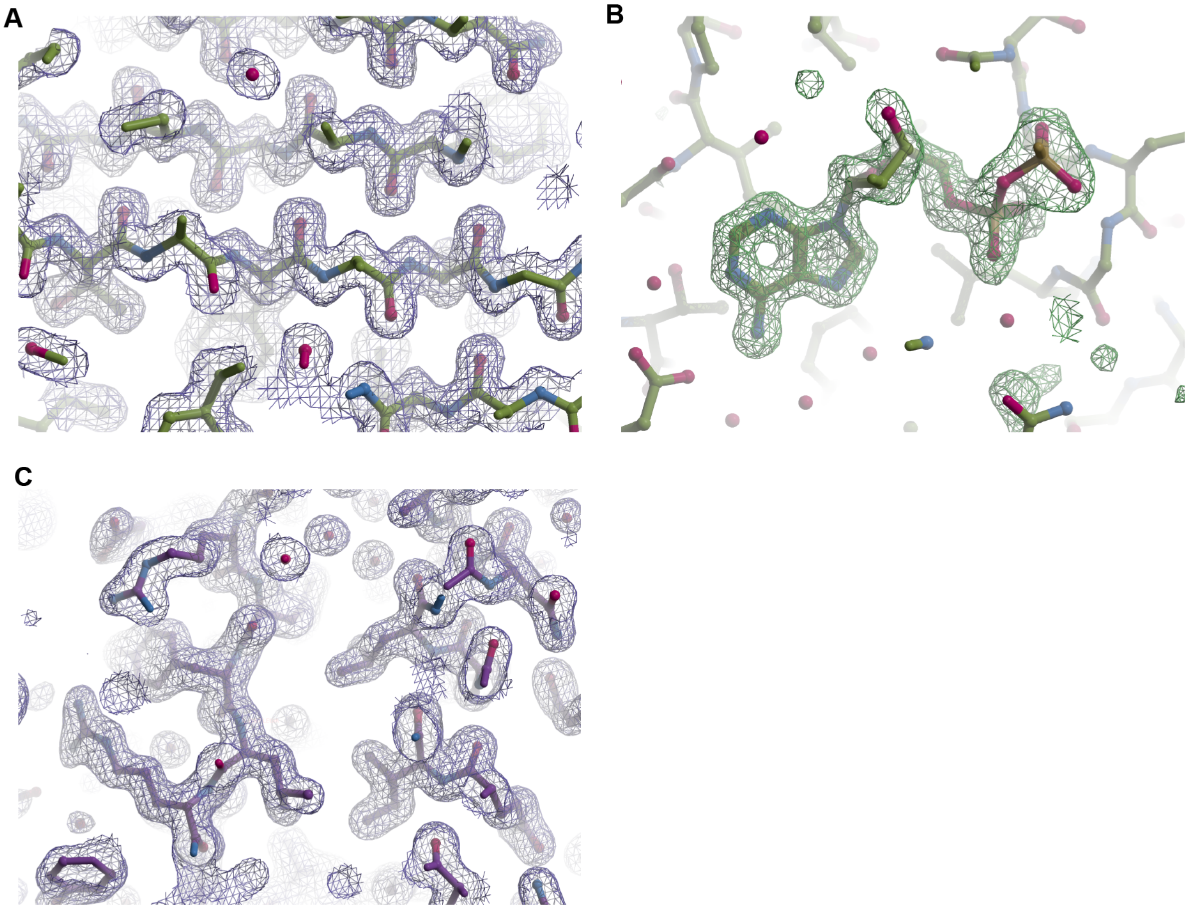

Supplementary Figure 14. X-ray crystallography of $LvrB_{CA}$:ADP and $LvrB_{CA}$ (A) Fourier density map ($2F_{obs}$-DF_{calc}) contoured at 1σ of a selected region of $LvrB_{CA}$:ADP (PDB 9QJG). (B) Omit map of ligand ADP:Mg^{2+} contoured at 3.5σ. (C) Fourier density map ($2F_{obs}$-DF_{calc}) contoured at 1σ of a selected region of $LvrB_{CA}$ (PDB 9QL9).

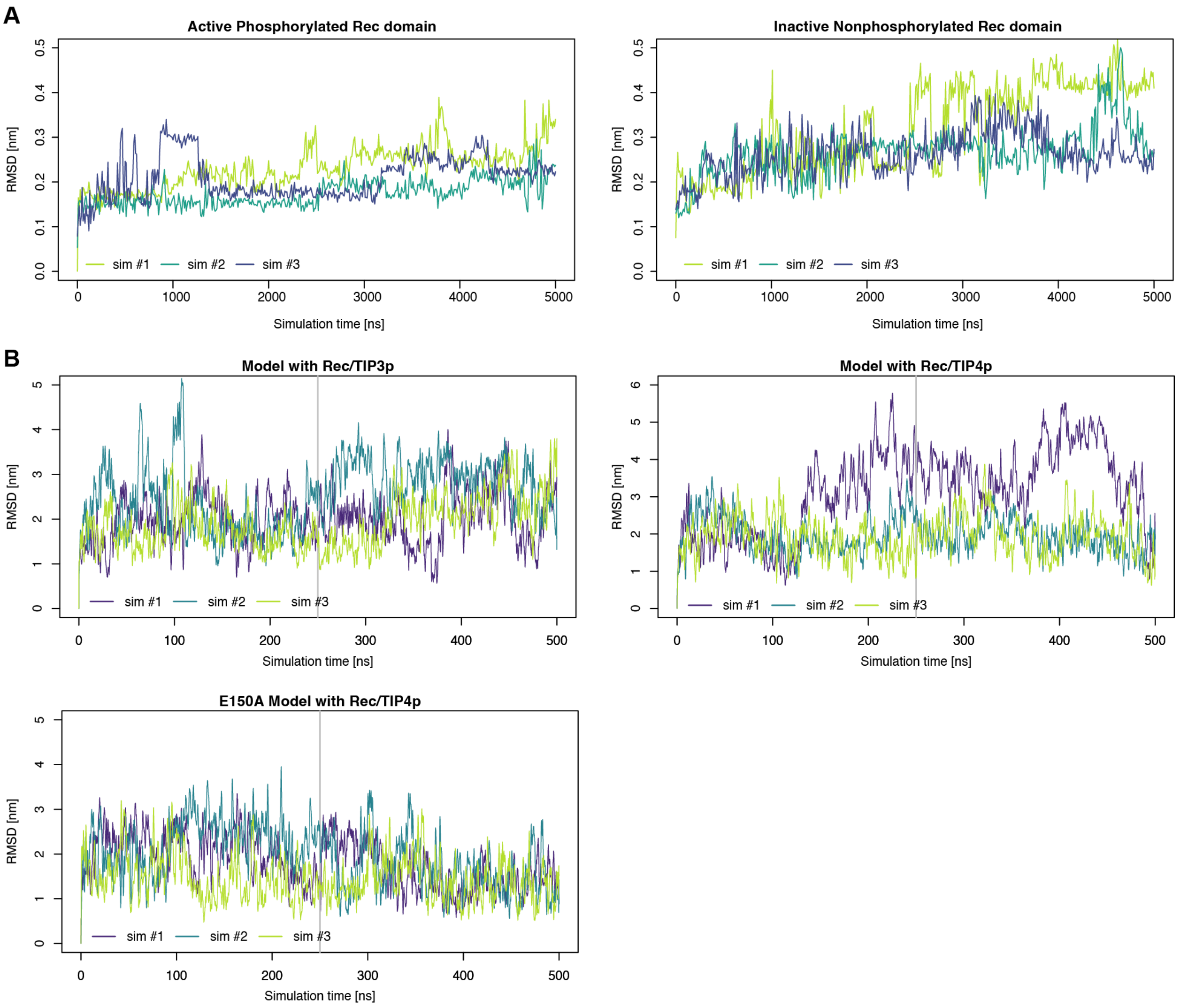

Supplementary Figure 15. (A) Time-resolved backbone Cα root mean square deviations (RMSD) of isolated Rec domains (K3–R129r) relative to the initial frame. The increase in RMSD in the second half of simulation #1 of nonphosphorylated Rec domain mainly stems from local unfolding of the artificially terminated helix α1. The local surroundings of Y87 and Y103 were not influenced. The simulation was therefore kept for calculating ensemble averages. (B) Time-resolved backbone root mean square deviations (RMSD) relative to the initial frame, obtained after the fit on the DHp domain (residues 150–213) from MD simulations of models under tension with Rec domain. The grey line at 250 ns indicates the time from which data collection for tilt distributions and coiled-coil distances (Fig. 4B,C and Supplementary Fig. 10) was initiated. The independent replicas were initiated from different configurations generated by independent pre-equilibration of the simulations. Simulation #1 in the simulation of TIP4p showed a temporary increase of RMSD corresponding to the expected knee-bending motion. Visual inspection showed that the protein structure was fully intact at all times.

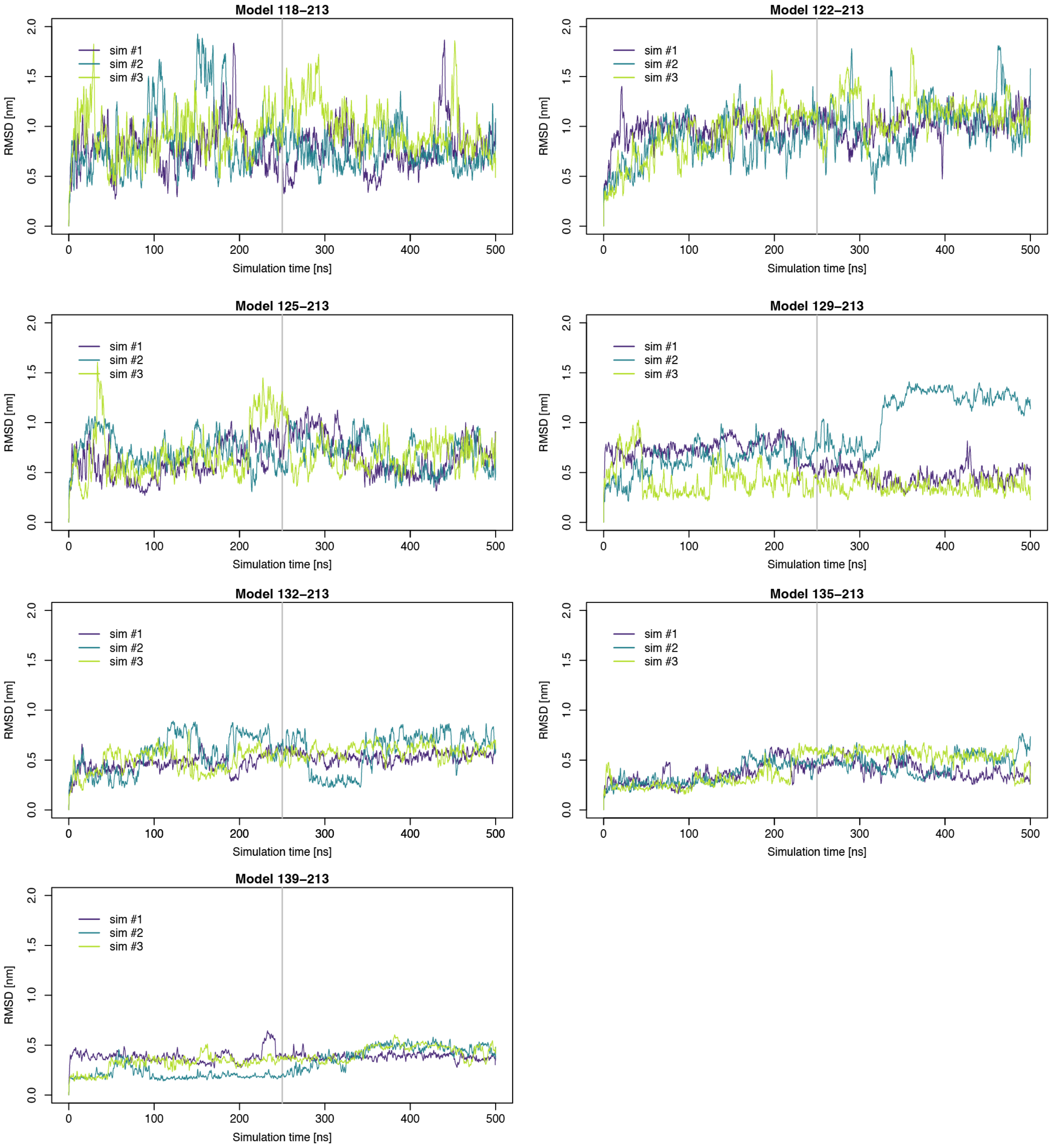

Supplementary Figure 16. Time-resolved backbone root mean square deviations (RMSD) relative to the initial frame, obtained after the fit on the DHp domain (residues 150–213) from MD simulations of models under tension with varying length. The grey line at 250 ns indicates the time from which data collection for tilt distributions and coiled-coil distances (Fig. 4B,C and Supplementary Fig. 10) was initiated. The independent replicas were initiated from different configurations generated by independent pre-equilibration of the simulations. Simulation #2 of model 129–213 was excluded from further analysis due to its non-equilibrium behavior.

Supplementary Tables

Supplementary Table 1. Cryo-EM statistics

	**LvrB:ATPγS, inactive** (PDB 9QR2)	**LvrB:ADP:BeF_3^-, activated** (PDB 9QQW)
**Data collection and processing**		
Microscope	TFS Glacios	TFS Glacios
Detector	Gatan K3 (6k x4k)	Gatan K3 (6k x4k)
Magnification	46’000	46’000
Voltage (kV)	200	200
Electron exposure ($e^-/Å^2$)	53–58	60
Defocus range (μm)	-1.0 to -2.0	-1.0 to -2.0
Pixel size (Å)	0.878	0.878
Symmetry imposed	C2	C1
Initial particle images (no.)	4,131,414	2,062,480
Final particle images (no.)	133,625	129,432
Map resolution (Å)	4.24	5.92
FSC threshold	0.143	0.143
Map resolution range (Å)	3.61–64.64	1.82–13.48
**Refinement**		
Initial model used	Rx model 8VC9 (this study)	Rx model 8VC9 (this study)
Model composition		
Chains	5	5
Non-hydrogen atoms	6018	3097
Protein residues	752	566
Water molecules	4	7
Ligands	ATPγS (2), Mg^{2+} (2)	ADP (2), Mg^{2+} (4), BeF_3^- (2)
R.m.s. deviations		
Bond lengths (Å)	0.006	0.018
Bond angles (°)	1.102	1.918
Validation		
MolProbity score	1.74	1.75
Clashscore	7.75	6.68
Rotamer outliers (%)	0.00	0.00
Ramachandran plot		
Favored (%)	95.45	94.38
Allowed (%)	4.55	5.62
Outliers (%)	0.00	0.00
Cb outliers (%)	0.00	1.11
Model to map correlation coefficients		
CCbox – whole map	0.93	0.90
CCmask – masked map	0.85	0.77

Supplementary Table 2. X-ray data collection and refinement statistics

	$LvrB_{CA}$	$LvrB_{CA}$:ADP	LvrB:AMPPCP:BeF_3^-	LvrC:ADP
**Data collection**				
Beamline	SLS X06SA	SLS X06SA	Soleil Proxima 1	SLS X06SA
Wavelength (Å)	1.000039	1.000039	0.978570	1.000040
Space group	$P2_12_12_1$	$P2_12_12_1$	C2	$P2_12_12_1$
Cell dimensions				
a, *b*, *c* (Å)	41.54, 57.15, 135.45	50.13, 54.90, 135.94	175.07, 102.33, 184.48	70.29, 85.21, 111.27
α, β, γ (°)	90.00, 90.00, 90.00	90.00, 90.00, 90.00	90.00, 92.49, 90.00	90.00, 90.00, 90.00
Resolution (Å)	43.68–1.50	42.71–1.60	49.27–2.65	55.64–2.60
R_{merge}	0.045	0.093	0.045	0.127
R_{pim}	0.013	0.027	0.028	0.056
Mean I / σI	25.3	26.2	14.4	9.0
Completeness (%)	99.7	99.9	99.8	99.9
Redundancy	13.2	13.2	4.2	6.3
**Refinement**				
Resolution (Å)	1.50	1.60	2.65	2.60
No. unique reflections	52536	50251	94249	21174
R_{work} / R_{free}	0.2259 / 0.2677	0.2040 / 0.2263	0.2124 / 0.2540	0.2142 / 0.2741
No. atoms (non-H)	2655	2830	18135	4810
Protein	2443	2490	17857	4674
Ligand/ion	0	54	242	71
Water	212	286	36	65
Average *B*-factor	37.66	20.31	101.49	55.22
R.m.s. deviations				
Bond lengths (Å)	0.007	0.006	0.006	0.009
Bond angles (°)	1.13	1.08	1.17	1.39
Ramachandran				
Favored (%)	93.88	97.35	97.86	94.72
Allowed (%)	5.78	2.65	2.00	4.26
Outliers (%)	0.34	0.00	0.13	1.02
PDB ID	9QL9	9QJG	8VC9	9QIH
