# Supplementary material for: Activation mechanism of the full-length histidine kinase LvrB from pathogenic Leptospira
Source: Nat Commun. 2026 Apr 16;17:5298. doi: 10.1038/s41467-026-71783-4 (PMC13269768; doi:10.1038/s41467-026-71783-4)
Supplement: Supplementary file 3 — Transparent Peer Review file [file 41467_2026_71783_MOESM3_ESM.pdf]

nature portfolio

Peer Review File

Activation mechanism of the full-length histidine kinase LvrB from pathogenic Leptospira

Corresponding Author: Professor Sebastian Hiller

This file contains all reviewer reports in order by version, followed by all author rebuttals in order by version.

Version 0:

Reviewer comments:

Reviewer #1

(Remarks to the Author)
Review: Activation mechanism of the full-length histidine kinase LvrB, a master virulence regulator in pathogenic Leptospira

This manuscript reports important findings regarding the two-component signaling (TCS) pathway in Leptospira, focusing on the histidine kinase LvrB. The authors have successfully resolved the full-length cryo-electron microscopy (cryo-EM) structures of LvrB in both its inactive and active conformations, and complemented these with X-ray, mutagenesis and NMR experiments, as well as molecular dynamics simulations.

Key contributions include the experimental determination of the inactive symmetric dimer, where catalytic domains are distant from the phosphorylatable histidine (H161), and the active asymmetric conformation characterized by a ~ 45o bend at the S–DHp junction. This conformational change involves a rearrangement in the REC domain, formation of a coiled-coil in the αS helix, and a switch in supercoil handedness. Furthermore, the study identifies an inverse Y–T switch mechanism in the REC domain and pinpoints the cognate response regulator (LvrC) through structural modeling and phosphotransfer assays, for which a X-ray structure is also provided. Molecular dynamics simulations suggest that coiled-coil formation alone is sufficient to induce the observed αS–DHp bending.

While these structural insights are valuable, the claims of explaining the full signaling mechanism appear speculative and are supported by limited experimental evidence. Moreover, despite providing a full-length structure, the study offers few fundamentally new insights into histidine kinase activation, as the principle of coiled-coil switching has been previously observed and characterized in fragments of other histidine kinase domains (Schmidt et al, 2016, https://doi.org/10.1002/pro.3095).

MAJOR POINTS:
1. The proposed mechanistic pathway for the activation of LvrB remains largely hypothetical (Figure 3). There is no direct experimental or computational evidence supporting the specific conformational steps depicted. To strengthen this aspect of the manuscript, the authors could perform metadynamics simulations (or other enhanced sampling approaches) to explore the transition from the inactive to the active state (or vice versa).

2. The MD simulations exclude the REC domain, which the authors propose as the driver of αS coiled-coil formation (through the 4-5 to 4-5-5 rearrangement). The authors could explore with molecular dynamics simulations whether including the REC domain in its active 4-5-5 conformation be sufficient to promote αS coiling and the associated bending and release of CA domain?

3. The paper suggests that E150 introduces repulsive strain at a heptad position, driving local unfolding and the 45° kink at the αS–DHp junction. Have the authors tested whether mutating E150 to alanine abolishes the kink or alters the unfolding pattern? This could also be tested computationally via mutation and MD simulation.

4. The authors describe local unfolding near L149–M152 and A156 as critical for the kink, but do not quantify this relationship. The authors could plot the time-resolved correlation between the bending angle at E150 and the dihedral angles of residues L149–M152 (one monomer) and A156 (the other monomer) during the MD trajectories.

5. The authors highlight a reversal of the canonical REC switch, with Y103 facing inward and T86 coordinating the phosphate. This is an interesting finding; how common is this inverse switch? Could the authors quantify this bioinformatically? Would molecular dynamics simulations of the isolated REC domain (phosphorylated and unphosphorylated) reveal how the position of Y103 influences T86 orientation and 4-5-5 formation?

6. The authors report using the CHARMM36 force field in combination with the TIP4P water model for their molecular dynamics simulations. However, CHARMM36 is specifically parameterized with the TIP3P water model (Best et. al, 2012, https://doi.org/10.1021/ct300400x), and substituting it with TIP4P without reparameterization may lead to inconsistencies in protein–solvent interactions and conformational stability. Generally, TIP4P-based models have been successfully applied to intrinsically disordered proteins to promote unfolded or flexible states (Piana et. al, 2015, https://doi.org/10.1021/acs.jctc.5b00255). Using TIP4P in conjunction with CHARMM36 for folded, structured systems should be carefully justified (as done in Huang et. al, 2017, https://doi.org/10.1038/nmeth.4067). The authors should either validate the impact of using TIP4P in their system or consider repeating key simulations with TIP3P to ensure consistency with the force field's original design.

7. In Figure S9, the observation that the coiled-coil unfolds in many simulations. Is this due to the TIP4P water model or other aspects of the modeling? Additionally, while the authors conclude that the heptad repeat is required, the coiled-coil appears to form only when an additional one to three turns are included (residues 118 to 129), which seems to contradict their conclusion or require further clarification.

8. In the Methods section, the authors state that the αS helix was modeled "as a straight elongation of the DHp domain in coiled-coil conformation" for molecular dynamics simulations. However, it remains unclear how the αS helix from the active state was connected to the DHp domain from the inactive state, especially given that the conformational mismatch at the αS–DHp junction is critical to the observed bending and unfolding. The authors should clarify whether the αS helix was taken directly from the active-state structure and grafted onto the DHp domain from the inactive state and how structural continuity and backbone geometry were resolved at the αS–DHp junction during model preparation.

9. The manuscript does not report the RMSD (root-mean-square deviation) of the system over time, nor does it indicate the duration of the production runs (500 ns or does this include equilibration?) or when equilibration was considered complete. Reporting RMSD is essential to assess the structural stability and convergence of the molecular dynamics simulations. The results for each replica should be reported separately.

MINOR POINTS:
10. The authors use ATPγS and BeF_3^- to mimic phosphorylated states. While clear for experts, a brief rationale for their use and the specific structural states they represent should be provided for broader clarity.

11. In Figure 2, the authors present the interaction details within the Rec domains for both the active (A) and inactive (B) states. However, the interaction diagrams (top row of the figure) are not aligned with the top-view structural representations (bottom row of the figure) of the Rec dimer shown for each state, which makes it challenging to locate the described interactions in the full 3D context.

12. Figure 5 presents a model for LvrB's function. While serving as a useful visual summary of the proposed signaling pathway, it largely depicts a canonical two-component system activation mechanism. It would be beneficial if the figure could more clearly highlight novel aspects unique to the LvrB system, or explicitly delineate experimentally supported steps from those that remain more speculative, aligning with the limitations of the provided experimental evidence

13. The presentation of experimental methods in the manuscript does not consistently follow the order of results. Additionally, the authors employ a generic "Bioinformatics" header to describe both server usage and the setup of molecular dynamics simulations, despite the latter being more accurately categorized as molecular modeling.

14. It is unclear why LvrB and LvrC were mixed for crystallization attempts when only LvrC's structure was ultimately determined (Section 'LvrC crystallization and structure determination', line 566), especially given that lines 234-236 state the complex did not crystallize.

15. The authors should characterize the difference in relative orientation of the CA domains between the active and inactive states (lines 103-105)

16. A clearer explanation of how the pathway was modeled is needed. (lines 161 - 162, Figure 3C and D)

17. In Figure S6, the authors should quantify the structural similarity between the REC, DHp, and CA domains derived from the cryo-EM and X-ray structures.

18. In Figure S9, Model 129-221, the first bead is not consistently highlighted in panel A and B. In panel B, it appears to be the first residue, which it should not be.

19. A detailed explanation of how the CA domain was modeled is required (lines 196-199 and Figure S10A).

Reviewer #2

(Remarks to the Author)
As part of two-component systems, sensor histidine kinases (HK) regulate diverse cellular responses throughout the bacterial kingdom. Despite the wide distribution and eminent roles of HKs, their signal-transduction mechanisms are still under vigorous debate. This is not least owing to the dearth of structural information on full-length HKs in both the presence and absence of the cognate signal. It is exactly this deficiency in our knowledge that the present contribution by Agustoni et al. seeks to address. Using cryo-EM, supplemented by X-ray diffraction analyses, structures of the Leptospira LvrB HK which uses a receiver domain (REC) as the input domain were solved in the unphosphorylated REC state and in the presence of the phosphomimic beryllium fluoride. Comparison of the two structures (albeit at relatively modest resolution) provides insight into the likely signaling mechanism – starting from a C2-symmetric conformation where the catalytically active CA domains are sequestered, REC phosphorylation goes along with a kink within the HK output module and liberation of one of the two CA domains. Biochemical analyses show enhanced kinase activity in this state. As another major result, the study identifies the downstream response regulator that LvrB acts upon. X-ray crystallography reveals the structure of this protein. Altogether, there can be little doubt that this is a stellar manuscript jam-packed with exciting results that significantly advance the field. The study is very well conceived, planned, executed, evaluated, and presented. Given the high relevance of the research and its quality, publication in Nat Commun is strongly advocated. All the below comments are fairly minor and can be addressed by the authors without need for further review.

Itemized list of remarks:
1. #27: it may be confusing to designate LvrB as a response regulator (which clearly it is) when the downstream partner LvrC can also be considered a response regulator
2. #30: throughout the ms., the authors designate the HK states as ‘active’ and ‘inactive’. However, it is well established that most HKs cycle between kinase- and phosphatase-active states. Is there any evidence to this effect for LvrB? At the very least, the authors should entertain the principal possibility of phosphatase activity in the so-called ‘inactive’ state.
3. #61: mention LvrA somewhere in the Introduction
4. #79: the appearance of two bands of similar intensity on the PhosTag gel is taken as evidence for hemi-phosphorylation. An alternative interpretation would be that a certain fraction of LvrB is inactive. Can this be ruled out?
5. #82: the determination of the LvrB structure is quite the feat, but the attained resolution leaves wanting. How confident are the authors regarding sidechain placement, esp. for the beryllium fluoride structure which has even poorer resolution?
6. #93: maybe mention that the CA arrangement could well change upon binding of the downstream partner LvrC
7. #109: sadly, the LvrB crystal structure adds very little. In fact, it raises a question in that beryllium fluoride was added, yet the structure corresponds to the unphosphorylated cryo-EM structure. Most likely, this owes to crystal packing but it warrants further comment, if ever so briefly.
8. #124: see above comment; how confident can the amino acids be modelled? is this mainyl based on the diffraction or the cryo-EM data, or both?
9. #170: specify which heptad position (a-g) E150 would end up in
10. #186: as the authors note, REC-HKs recur in the databases. Can the modelling results be correlated with an analysis of the linker lengths of the available REC-HKs?
11. #208: more precisely, the model is ‘probed’ rather than ‘validated’ by the mutagenesis experiments
12. #243: why use the designation alpha4-beta5-alpha5 here when before 4-5-5 was used?
13. fig. 1E needs some explanation how the two structures were aligned and colored. Only much later in the paper it turns out that this is based on the modeling which seems to predict a larger phosphorylation-induced rearrangement than apparent at first glance. It may be safer to represent the structure in panel E turned by around 180° around the vertical axis.
14. fig. 3C: in the left-most cartoon, the gray protomer seems to have one green residue which should be gray as well

Reviewer #3

(Remarks to the Author)
The manuscript by Agustoni et al. addresses the activation mechanism of a small sensory histidine kinase LvrB, which serves as a master regulator in the pathogen Leptospira. LvrB only consists of two domains: the N-terminal Rec domain responds to an upstream phosphorylation signal, which in turn activates the C-terminal histidine kinase via auto-phosphorylation. Despite the relatively small size, the authors have obtained the cryoEM structures of the full-length LvrB in two distinct dimeric forms, representing the kinase-inactive and kinase-activated states, respectively. Based on structural comparisons, the authors propose that the kinase activation entails significant rearrangement in signaling helices involving the coiled-coil formation at the dimer interface and breaking of dimer symmetry. As intriguing as these findings are for structural studies of LvrB, this proposal is not necessarily groundbreaking for sensory histidine kinases. Several cryoEM and crystal structures have been reported for full-length HKs in dimeric forms, suggesting a general strategy of allostery shared among sensory histidine kinases, although the mechanistic details remain elusive. A major challenge is to figure out how the allosteric action propagates through a dimeric framework, for which LvrB perhaps represents the best minimalist model for structural dissection. To this end, the work is deemed to fall short of expectation. Given ~2 millions of particles picked for LvrB prepared with and without BeF_3 (a phosphorylation mimic), the two structures presented in this work were reconstructed from <10% particles, which begs some questions regarding the rigor of data analysis. Are there additional states other than these two reported? How flexible or dynamic are the LvrB molecules in solution? Is there any population of LvrB particles that allude to the splayed conformational state revealed by crystallographic studies? Overall, the topic of this work is significant, and the results are interesting. However, its publication in Nat. Comm. may require revision of the dimeric model in the inactive state (see below) as well as additional work based on more inclusive and in-depth particle analysis based on the advanced algorithms such as 3DVA and 3DFlex of cryoSPARC.

Major:
1) Based on the view in Fig. 1D – the handedness of the inactive dimer is inconsistent between the cryoEM map (left) and model (right). Specifically, the map clearly shows a dimer in a left-handed twist while the model shown in ribbon diagram seems to adopt a right-handed twist.

2) Given the limited resolution of the reported cryoEM maps for both states (4.2Å and 5.9 Å, respectively), it is important to include a figure showing a side-by-side map comparison in the signaling helix regions as shown in Fig. 3c and Fig. S3H.

3) The proposal of the inverse Y-T switch in the REC domain is intriguing. Again, a side-by-side map comparison in the same region between two states would be informative. Does the inverse Y-T switch apply to both protomers? Is breaking of dimer symmetry also observed in the REC domain. In some REC-based sensory proteins, the Y-T switch may only take place in one of two promoters upon pseudo-phosphorylation (dimer asymmetry).

4) What is the mode of auto-phosphorylation for LvrB? trans or cis? Fig. 5 suggests a mode of cis-phosphorylation. Is there any additional supporting (biochemical) data for this proposed cis- mode beyond the structural data? The information may help resolve possible ambiguity at the crossing of DHp and CA domains given the limited resolution of the activated state.

5) Regarding the hemi-phosphorylation, the kinase activity assays presented in Fig. 1B and Fig. 1C do not seem to support this notion. While Fig. 1C (Phos-tag based) appears to render a 1:1 ratio in intensity between the phosphorylated (top) band and unphosphorylated (bottom) band, Fig. 1B (32P-based) shows a clear increase in the phosphorylated signal as a function of the reaction time while the total protein band of LvrB remains largely constant. Please explain.

Reviewer #4

(Remarks to the Author)
This manuscript presents the first 3D structures of both the activated and inactivated states of a full-length histidine kinase, LvrB, from Leptospira. LvrB is a Rec-controlled histidine kinase, a class of regulatory proteins central to bacterial two-component signaling systems.

This study represents a significant advance, combining single-particle cryo-electron microscopy and X-ray crystallography to elucidate key structural transitions associated with LvrB activation.

In the autokinase-inactive state, LvrB adopts a symmetric conformation, with both unphosphorylated receiver (Rec) domains maintaining the separation of the αS helices.

The authors also report a structure of the activated state, mimicked using BeF_3^-, ADP, and Mg^{2+}.

While the cryo-EM reconstruction is at relatively low resolution, a clear rearrangement of the Rec domains is observed, which is independently confirmed by well-resolved X-ray crystallographic data.

The resolution of both conformational states allows the authors to propose a mechanistic model of activation: Rec domain rearrangement leads to coiled-coil formation of the αS helices, release of the CA (catalytic and ATP-binding) domain, and ultimately, phosphorylation of LvrC, a newly identified downstream response regulator.

These findings contribute important structural insight into a signaling pathway that has been largely unexplored at the molecular level.

Overall, the study is technically rigorous, with comprehensive datasets and a well-supported interpretation.

The methods are clearly described, and the integration of cryo-EM with crystallography is a notable strength.

This work represents a substantial contribution to the field and advances our understanding of Rec-controlled histidine kinases and two-component systems.

In conclusion, this is an excellent and impactful study, and I support its publication.

Reviewer #5

(Remarks to the Author)
I co-reviewed this manuscript with one of the reviewers who provided the listed reports. This is part of the Nature Communications initiative to facilitate training in peer review and to provide appropriate recognition for Early Career Researchers who co-review manuscripts.

Reviewer #6

(Remarks to the Author)
I co-reviewed this manuscript with one of the reviewers who provided the listed reports. This is part of the Nature

Communications initiative to facilitate training in peer review and to provide appropriate recognition for Early Career Researchers who co-review manuscripts.

Version 1:

Reviewer comments:

Reviewer #1

(Remarks to the Author)
The authors have addressed the previous concerns. The manuscript has been significantly improved. However, a few minor technical points and formatting issues should be resolved prior to publication.

Figure 4: In panels C and D, please add direct labels or annotations to the figure itself to distinguish between the modelled and experimental structures. Relying solely on the figure caption for this distinction makes the data more difficult for the reader to parse quickly.

Figure S14 & S15 (Stability Analysis): The RMSD plots indicate that certain replicas have not reached convergence (specifically Figure S14: Panel A, simulation #1 (inactive); Panel B, simulation #1 with TIP4P; and Figure S15: model 129-211, simulation #2). Given that three replicas were performed for each system, the authors should consider excluding these specific non-converged trajectories from the final calculated averages or downstream analysis to ensure the reported statistics reflect a stable equilibrium. At a minimum, the text should explicitly acknowledge these divergences and justify their inclusion.

Line 675: Change "Refmac" to the standard nomenclature REFMAC5.

Reviewer #3

(Remarks to the Author)
The revised manuscript has largely addressed the concerns raised in the last round. It is well written with much improved clarity. In addition, the data is clearly presented, solid and comprehensive. While a few aspects of the mechanism remain unresolved, this work nevertheless represents a major advance on a highly interesting topic relevant to bacterial signaling. I support its publication in Nat. Comm. with minor changes. See below.

1) If possible, I suggest to present the crystal structure of LvrB in main figures in lieu of Fig. S6. Even though it is unknown at the moment how this “splayed” pseudo-phosphorylated dimer structure fits in the grand scheme of the LvrB activation, it represents a unique conformational state, perhaps an intermediate state trapped in crystal lattice as stated in Line 122-124. It is curious whether the third state in the proposed activation mechanism (Fig. 3C) is based on this crystal structure. To this end, it would be helpful for readers to gauge the tethering and handedness of the linker helices at the dimer interface if the two protomers are colored differently in Fig. S3B.
2) The importance of E150 at the aS/DHp junction is noted. Yet, no mutational data on E150 is shown to support this. Any explanation?
3) With a “liberated” (in other words “unresolved”) CA domain, one would take caution in assigning this asymmetric state to the “kinase-active” state. Based on Fig. 6, it is more compatible with a “phosphoryltransferase”-competent state. This circles back to my previous questions regarding the particle picking/filtering strategy. While it is indeed a common practice to use only a small fraction of particles (4% of total particles picked in Fig. S5; and <10% in Fig. S3) for 3D construction and refinement, it is not necessarily the best practice, particularly in this case where different conformational states of varying population are important for mapping out the trajectory of the kinase activation. An inclusive strategy that explores all possible states is critical. I also understood that such a task is beyond the scope of this work.

Open Access This Peer Review File is licensed under a Creative Commons Attribution 4.0 International License, which permits use, sharing, adaptation, distribution and reproduction in any medium or format, as long as you give appropriate credit to the original author(s) and the source, provide a link to the Creative Commons license, and indicate if changes were made.
In cases where reviewers are anonymous, credit should be given to 'Anonymous Referee' and the source.
The images or other third party material in this Peer Review File are included in the article's Creative Commons license, unless indicated otherwise in a credit line to the material. If material is not included in the article's Creative Commons license and your intended use is not permitted by statutory regulation or exceeds the permitted use, you will need to obtain permission directly from the copyright holder.
To view a copy of this license, visit https://creativecommons.org/licenses/by/4.0/

Reviewer comments in black – our response in blue:
Reviewer #1:

This manuscript reports important findings regarding the two-component signaling (TCS) pathway in Leptospira, focusing on the histidine kinase LvrB. The authors have successfully resolved the full-length cryo-electron microscopy (cryo-EM) structures of LvrB in both its inactive and active conformations, and complemented these with X-ray, mutagenesis and NMR experiments, as well as molecular dynamics simulations.

Key contributions include the experimental determination of the inactive symmetric dimer, where catalytic domains are distant from the phosphorylatable histidine (H161), and the active asymmetric conformation characterized by a ~ 45° bend at the S–DHp junction. This conformational change involves a rearrangement in the REC domain, formation of a coiled coil in the αS helix, and a switch in supercoil handedness. Furthermore, the study identifies an inverse Y–T switch mechanism in the REC domain and pinpoints the cognate response regulator (LvrC) through structural modeling and phosphor-transfer assays, for which a X-ray structure is also provided. Molecular dynamics simulations suggest that coiled-coil formation alone is sufficient to induce the observed αS–DHp bending.

While these structural insights are valuable, the claims of explaining the full signaling mechanism appear speculative and are supported by limited experimental evidence. Moreover, despite providing a full-length structure, the study offers few fundamentally new insights into histidine kinase activation, as the principle of coiled-coil switching has been previously observed and characterized in fragments of other histidine kinase domains (Schmidt et al, 2016, https://doi.org/10.1002/pro.3095).

We thank the referee for their appreciation of our work. We fully agree that the principle of coiled-coil switching has been nicely reviewed by Schmidt et al. This study is now being cited in the revised manuscript and Figure 3A has been extended to include the heptad repeat of DHp α1. LvrB features a 3-residue insertion between the coiled coils in the αS helix and the DHp bundle, which corresponds to an insertion index $I_A = –0.5$ in the nomenclature of Schmidt et al.
Compared to previous work, we provide the first set of structures of a full-length natural Rec-controlled HK in both the active and inactive state, allowing us to discern the mechanism in unprecedented detail.

MAJOR POINTS:
1. The proposed mechanistic pathway for the activation of LvrB remains largely hypothetical (Figure 3). There is no direct experimental or computational evidence supporting the specific conformational steps depicted. To strengthen this aspect of the manuscript, the authors could perform meta-dynamics simulations (or other enhanced sampling approaches) to explore the transition from the inactive to the active state (or vice versa).

The transition between the two end points – which are experimentally determined structures – was inferred by helix modelling. With the assumption that the DHp bundle does not dissociate on the timescale of the transition, the modeled transition pathway is the only biophysically plausible way to connect the end structures. We have clarified this reasoning in the revised manuscript.

We fully agree that monitoring this transition in finer detail would be valuable. We evaluated the possibility of performing meta-dynamics simulations; however, this would be a non-trivial undertaking requiring substantial computational and personnel resources that are unfortunately beyond the scope of the current manuscript.

2. The MD simulations exclude the REC domain, which the authors propose as the driver of αS coiled-coil formation (through the 4-5 to 4-5-5 rearrangement). The authors could explore with molecular dynamics simulations whether including the REC domain in its active 4-5-5 conformation be sufficient to promote αS coiling and the associated bending and release of CA domain?

We thank the reviewer for this suggestion. We have performed the suggested MD simulations. The results were essentially similar to the simulation with a long helix (residues 118–213): the bending of the coiled coils was observed, confirming that the Rec domains in the 4-5-5 arrangement indeed stabilize the αS coiled coil. The observed bending angle was even slightly increased. These new data are now shown in Fig. 4.

3. The paper suggests that E150 introduces repulsive strain at a heptad position, driving local unfolding and the 45° kink at the αS–DHp junction. Have the authors tested whether mutating E150 to alanine abolishes the kink or alters the unfolding pattern? This could also be tested computationally via mutation and MD simulation.

We thank the reviewer for this suggestion. We have performed MD simulations of the E150A mutant (N=3, 500ns each, including the Rec domains). These simulations show that the kinking angle decreases drastically upon the mutation E150A, in full agreement with our hypothesis that E150 contributes to destabilizing the straight conformation through electrostatic repulsion. These new data are now included in Fig. 4.

4. The authors describe local unfolding near L149–M152 and A156 as critical for the kink, but do not quantify this relationship. The authors could plot the time-resolved correlation between the bending angle at E150 and the dihedral angles of residues L149–M152 (one monomer) and A156 (the other monomer) during the MD trajectories.

We thank the reviewer for this suggestion. The time trajectory of secondary structure and tilt angle are now illustrated in Supplementary Figure S10, for a simulation including the Rec domains (TIP4P water model). We analyzed the secondary structure of residues E141 to M159 using DSSP (Kabsch and Sander, Biopolymers **22**, 2577 (1983)). The data show a clear correlation between local unfolding and bending. Furthermore, the analysis reveals the asymmetric behavior of the dimer – each chain undergoes local secondary structure changes in a different manner.

5. The authors highlight a reversal of the canonical REC switch, with Y103 facing inward and T86 coordinating the phosphate. This is an interesting finding; how common is this inverse switch? Could the authors quantify this bioinformatically? Would molecular dynamics simulations of the isolated REC domain (phosphorylated and unphosphorylated) reveal how the position of Y103 influences T86 orientation and 4-5-5 formation?

We bioinformatically analyzed 177 000 predicted Rec domains from the UniProt data base. Less than 1% feature all the amino acids involved in the inverse Y-T switch, indicating this to be a rare yet distinctive element. We now mention this in the revised text.
As suggested by the reviewer, we have analyzed the positioning of side chains in two MD simulation sets of an isolated Rec domain, starting from either the active/phosphorylated or the inactive/non-phosphorylated conformation. For each condition, 3 simulations of 5µs each were performed, to capture the large-amplitude motions of Y103 and Y87. The analysis reveals an anticorrelation between the two tyrosines: only one occupies the binding pocket at any given time, with transitions between the two states. These new data are now shown in Fig. 2C,D and the Supplementary Figure S8A.

6. The authors report using the CHARMM36 force field in combination with the TIP4P water model for their molecular dynamics simulations. However, CHARMM36 is specifically parameterized with the TIP3P water model (Best et. al, 2012, https://doi.org/10.1021/ct300400x) and substituting it with TIP4P without reparameterization may lead to inconsistencies in protein–solvent interactions and conformational stability. Generally, TIP4P-based models have been successfully applied to intrinsically disordered proteins to promote unfolded or flexible states (Piana et. al, 2015, https://doi.org/10.1021/acs.jctc.5b00255). Using TIP4P in conjunction with CHARMM36 for folded, structured systems should be carefully justified (as done in Huang et. al, 2017, https://doi.org/10.1038/nmeth.4067). The authors should either validate the impact of using TIP4P in their system or consider repeating key simulations with TIP3P to ensure consistency with the force field's original design.

We fully agree with the reviewer that water models may influence protein behavior. The selection of TIP4p was based on our extensive previous experience simulating membrane proteins (Korn and Pluhackova, *Commun Chem* **8**, 38 (2025), Beigl et al., *EMBO Rep.* **25**, 3896 (2024), Degen et al. *Nature* **618**, 1065 (2023), Mari et al. *Nat. Comm.* **13**, 2609 (2022), Laskowski et al. *Nat. Comm.* **12**, 7082 (2021), Pluhackova et al. Front. Cell Dev. Biol. 9, 807913 (2021)), as well as previous work by others (Nutt and Smith, *J. Chem. Theory Comput.* **3**, 1550 (2007)). In that work, mTIP3p (CHARMM water with Lennard-Jones parameters on hydrogens), TIP4p and TIP5p were tested with the CHARMM force field. TIP4p gave the best agreement for free energy of solvation, and proteins remained stable in either of mTIP3p, TIP4p and TIP5p water.
Notably, the manuscript referred to by the referee (https://doi.org/10.1021/acs.jctc.5b00255), is not from Piana et. al, 2015, and no TIP4p is mentioned. We suppose that the referee meant Piana et al https://pubs.acs.org/doi/10.1021/jp508971m, or Piana et al (https://pubs.acs.org/doi/10.1021/acs.jctc.9b00251), where a new water model titled TIP4p-D was parametrized and assessed for studies of disordered proteins. Indeed, the stronger dispersion term in the TIP4p-D water model stabilizes disordered proteins, but this is novel to TIP4p-D and not a general feature of the original TIP4p used in our manuscript.
Nevertheless, as suggested, we have performed two batches of test simulations, once model 118-213 and once model with Rec domains (N=3 per condition, 500ns each) using the standard CHARMM TIP3p water model (sometimes also called mTIP3p). The comparison shows that the proteins behave similarly in TIP4p and TIP3p (Review-

only-Figure 1). If anything, the protein is somewhat more stably folded in TIP4p than in TIP3p. This confirms that our conclusions are robust to the choice of water model.

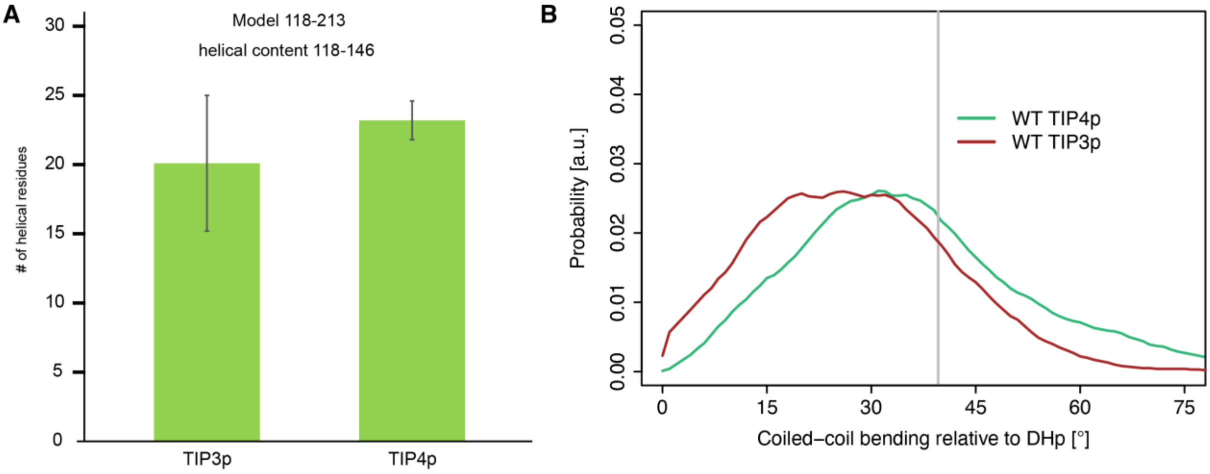

Review-only Figure 1. (A) Coiled coil unfolding measured as the average number of helical residues among the coiled coil residues 118–146 in MD simulations of the model spanning residues 118–213 either in CHARMM TIP3p or in TIP4p water model. Analysis is done in between 250 and 500 ns of simulation time for each chain separately, N=3 replicas per simulation type, resulting in 6 samples. The error bars denote standard deviation over the 6 values. **(B)** Impact of the water model (TIP4p vs. CHARMM TIP3p) on the bending of the coiled-coil domain relative to the DHp domain. To exclude the impact of the unfolding of the helix termini, quantified in subfigure A, the simulation of the model with Rec domains (residues 3–213) are analyzed.

7.In Figure S9, the observation that the coiled coil unfolds in many simulations. Is this due to the TIP4P water model or other aspects of the modeling?

It is typical in MD simulations that helices unfold from their ends (https://peerj.com/articles/4769/). Some degree of unfolding is thus expectable, mainly in the shortened coiled-coils. To exclude destabilizing effect of the water model, we have repeated the simulations of the model 118–213 in CHARMM TIP3p water (N=3, 500ns each, analysis over 250-500 ns for each chain individually). In CHARMM TIP3p water 20.1±4.9 residues of the coiled-coils spanning residues 118-146 were helical, in TIP4p the same analysis revealed 23.2±1.4 helical residues (Review-only Figure 1A). Thus, the unfolding of the ends of the coiled-coils is not due to the TIP4p water model.

Additionally, while the authors conclude that the heptad repeat is required, the coiled coil appears to form only when an additional one to three turns are included (residues 118 to 129), which seems to contradict their conclusion or require further clarification.

The unfolding of coiled coils is seen in both water models, so it is not an artifact of the method chosen, but an effect resulting from the absence of the Rec domain. Figure S10 shows that the coiled coil is the more stable the longer it is, which is what one would expect, as each heptad repeat adds a certain amount of ΔG to the stability. When elongating the helix, as soon as this stability is sufficiently large, it allows

overcoming the free energy penalty needed to kink the helices, and that is what we observe in the simulations.

8. In the Methods section, the authors state that the αS helix was modeled "as a straight elongation of the DHp domain in coiled-coil conformation" for molecular dynamics simulations. However, it remains unclear how the αS helix from the active state was connected to the DHp domain from the inactive state, especially given that the conformational mismatch at the αS–DHp junction is critical to the observed bending and unfolding. The authors should clarify whether the αS helix was taken directly from the active-state structure and grafted onto the DHp domain from the inactive state and how structural continuity and backbone geometry were resolved at the αS–DHp junction during model preparation.

Thanks for pointing this out - we have included a precise description how the model was created to the methods section.

9. The manuscript does not report the RMSD (root-mean-square deviation) of the system over time, nor does it indicate the duration of the production runs (500 ns or does this include equilibration?) or when equilibration was considered complete. Reporting RMSD is essential to assess the structural stability and convergence of the molecular dynamics simulations. The results for each replica should be reported separately.

We thank the reviewer for pointing this out. For all analyses, data acquisition was performed over the 250–500 ns time window; the initial 0–250 ns equilibration period was excluded. We now report the RMSD plots for all replicas of all simulations in the new Supplementary Figure S14 and S15.

<u>MINOR POINTS:</u>

10.The authors use ATPγS and BeF□$^-$ to mimic phosphorylated states. While clear for experts, a brief rationale for their use and the specific structural states they represent should be provided for broader clarity.

Thanks for pointing this out. We have included such rationale for each compound used.

11.In Figure 2, the authors present the interaction details within the Rec domains for both the active (A) and inactive (B) states. However, the interaction diagrams (top row of the figure) are not aligned with the top-view structural representations (bottom row of the figure) of the Rec dimer shown for each state, which makes it challenging to locate the described interactions in the full 3D context.

We have redrawn Figure 2 accordingly to match the details and include Y103 and Y87 in panel B.

12. Figure 5 presents a model for LvrB's function. While serving as a useful visual summary of the proposed signaling pathway, it largely depicts a canonical two-component system activation mechanism. It would be beneficial if the figure could more clearly highlight novel aspects unique to the LvrB system, or explicitly delineate

experimentally supported steps from those that remain more speculative, aligning with the limitations of the provided experimental evidence.

Figure 5 (now Figure 6) summarizes the mechanism and the downstream contact to LvrC, which has been established in the previous Figures in detail. This is a summarizing Figure and we feel it is important to keep as is for clarity to the reader.

13.The presentation of experimental methods in the manuscript does not consistently follow the order of results. Additionally, the authors employ a generic "Bioinformatics" header to describe both server usage and the setup of molecular dynamics simulations, despite the latter being more accurately categorized as molecular modeling.

Thanks for pointing this out, we have re-arranged and adapted the section headings accordingly.

14. It is unclear why LvrB and LvrC were mixed for crystallization attempts when only LvrC's structure was ultimately determined (Section 'LvrC crystallization and structure determination', line 566), especially given that lines 234-236 state the complex did not crystallize.

Indeed, LvrB and LvrC were mixed in an attempt to co-crystallize the LvrB–LvrC complex. The resulting crystals happened to contain only LvrC, while LvrB stayed in solution. Nonetheless, these allowed us the structure determination of LvrC. We have clarified the wording accordingly.

15. The authors should characterize the difference in relative orientation of the CA domains between the active and inactive states (lines 103-105)

We have clarified this in more detail.

16. A clearer explanation of how the pathway was modeled is needed. (lines 161 - 162, Figure 3C and D)

We have clarified this by adding text.

17. In Figure S6, the authors should quantify the structural similarity between the REC, DHp, and CA domains derived from the cryo-EM and X-ray structures.

We have included a direct comparison of the Rec domains to Figure S6.

18. In Figure S9, Model 129-221, the first bead is not consistently highlighted in panel A and B. In panel B, it appears to be the first residue, which it should not be.

Thanks for spotting this error. These pictures do no longer appear in the revised manuscript.

19. A detailed explanation of how the CA domain was modeled is required (lines 196-199 and Figure S10A).

The model was based on a homologous structure. We have clarified this now in the text.

Reviewer #2 (Remarks to the Author):
As part of two-component systems, sensor histidine kinases (HK) regulate diverse cellular responses throughout the bacterial kingdom. Despite the wide distribution and eminent roles of HKs, their signal transduction mechanisms are still under vigorous debate. This is not least owing to the dearth of structural information on full-length HKs in both the presence and absence of the cognate signal. It is exactly this deficiency in our knowledge that the present contribution by *Agustoni et al*. seeks to address. Using cryo-EM, supplemented by X-ray diffraction analyses, structures of the Leptospira LvrB HK which uses a receiver domain (REC) as the input domain were solved in the unphosphorylated REC state and in the presence of the phosphomimic beryllium fluoride. Comparison of the two structures (albeit at relatively modest resolution) provides insight into the likely signaling mechanism – starting from a C2-symmetric conformation where the catalytically active CA domains are sequestered, REC phosphorylation goes along with a kink within the HK output module and liberation of one of the two CA domains. Biochemical analyses show enhanced kinase activity in this state. As another major result, the study identifies the downstream response regulator that LvrB acts upon. X-ray crystallography reveals the structure of this protein.

Altogether, there can be little doubt that this is a stellar manuscript jam-packed with exciting results that significantly advance the field. The study is very well conceived, planned, executed, evaluated, and presented. Given the high relevance of the research and its quality, publication in Nat Commun is strongly advocated. All the below comments are fairly minor and can be addressed by the authors without need for further review.

We thank the reviewer for the positive assessment of our work and shared excitement.

Itemized list of remarks:

1.#27: it may be confusing to designate LvrB as a response regulator (which clearly it is) when the downstream partner LvrC can also be considered a response regulator

Indeed, LvrB is at the same time a response regulator and a histidine kinase. To avoid confusion, we have replaced “response regulator” in the abstract with “histidine kinase”, and added a sentence in the introduction to clarify this point.

2.#30: throughout the ms., the authors designate the HK states as ‘active’ and ‘inactive’. However, it is well established that most HKs cycle between kinase- and phosphatase-active states. Is there any evidence to this effect for LvrB? At the very least, the authors should entertain the principal possibility of phosphatase activity in the so-called ‘inactive’ state.

We fully agree with the reviewer that most HKs exhibit both kinase and phosphatase activities, typically segregated between different conformational states. We have clarified in text that by ‘active’ and ‘inactive’ we mean ‘kinase-active’ and ‘kinase-inactive’ states, therefore not excluding the possibility that the ’kinase-inactive’ state might be ‘phosphatase-active’. Furthermore, we have revised the Discussion to

explicitly address this point, proposing that the asymmetric state is kinase-active while the symmetric state may serve as the phosphatase-active form.
We have preliminary experimental data supporting that LvrB acts as an LvrC-specific phosphatase in its symmetric (kinase-inactive) conformation. However, a thorough characterization of all three LvrB catalytic activities – autokinase, phosphotransferase, and phosphatase – is beyond the scope of the current manuscript and will be reported in a future publication.

3.#61: mention LvrA somewhere in the Introduction

We have added this.

4.#79: the appearance of two bands of similar intensity on the PhosTag gel is taken as evidence for hemi-phosphorylation. An alternative interpretation would be that a certain fraction of LvrB is inactive. Can this be ruled out?

We thank the reviewer for pointing out this alternative interpretation. Di-phosphorylation (phosphorylation of both protomers) has been observed in other HKs under certain conditions (Bouillet et al. 2020, 10.1074/jbc.RA120.012757). In principle, our data would also be in agreement with a scenario where e.g. 50% of the protein material is completely inactive and the other 50% are active and di-phosphorylate. However, the likelihood for obtaining the same amount of inactive and dephosphorylation-active material by pure chance seems small, and the same result has been obtained with other HKs in completely different context (e.g. Kitanishi et al. https://doi.org/10.1074/jbc.M111.274811). Hence, our conclusion is most likely true.

5.#82: the determination of the LvrB structure is quite the feat, but the attained resolution leaves wanting. How confident are the authors regarding sidechain placement, esp. for the beryllium fluoride structure which has even poorer resolution?

We fully agree with this note of caution. Side chain placement was done based on high-resolution crystal structures. We explain this in detail in the Methods section.

6.#93: maybe mention that the CA arrangement could well change upon binding of the downstream partner LvrC.

Thanks, we are now mentioning this. Notably, LvrC can be docked onto activated LvrB without steric clashes (Fig. 6).

7.#109: sadly, the LvrB crystal structure adds very little. In fact, it raises a question in that berylliumfluoride was added, yet the structure corresponds to the unphosphorylated cryo-EM structure. Most likely, this owes to crystal packing, but it warrants further comment, if ever so briefly.

The crystal structure contributed valuable information, but perhaps we did not communicate this clearly enough. The structure did ***not*** correspond to the unphosphorylated cryo-EM structure, as the Rec domains are splayed apart. It had several merits:

- It provided an intermediate state that separated Rec activation from knee bending. This important intermediate thus shows that the Rec-Rec dimer is only loosely packed.
- It also provided an independent data point in the conformational space of LvrB. By overpopulating an intermediate state through crystal contacts, it delivers an additional view on the conformational landscape of LvrB that is not accessible by cryo-EM.
- Due to its high resolution, it delivered the details on the inverse YT switch and resolved the αS / CA interaction. Furthermore, the crystal structure was very helpful to determine side chain orientations at atomic resolution.

We have tried to make these points now clearer in the manuscript.

8. #124: see above comment; how confident can the amino acids be modelled? is this mainly based on the diffraction or the cryo-EM data, or both?

Indeed, side chain modeling was mainly based on the high-resolution diffraction data. This is described in detail in the model building part of the methods section.

9. #170: specify which heptad position (a-g) E150 would end up in.

For a straight helix, residue E150 would occupy the 'a' position. This is now mentioned in the text.

10. #186: as the authors note, REC-HKs recur in the databases. Can the modelling results be correlated with an analysis of the linker lengths of the available REC-HKs?

We have added such analysis in Supplementary Figure S4. The lengths of αS helices indeed have multiples of seven strongly overpopulated, as expected for heptad repeats.

11. #208: more precisely, the model is 'probed' rather than 'validated' by the mutagenesis experiments.

We have reworded.

12.#243: why use the designation alpha4-beta5-alpha5 here when before 4-5-5 was used?

Thanks, has been fixed.

13. fig. 1E needs some explanation how the two structures were aligned and colored. Only much later in the paper it turns out that this is based on the modeling which seems to predict a larger phosphorylation-induced rearrangement than apparent at first glance. It may be safer to represent the structure in panel E turned by around 180° around the vertical axis.

Thanks for pointing this out. We have matched the rotations of panels D and E by rotating the structure in panel D by 180° (rotating panel E would have hidden the knee behind the immobilized CA domain). The Rec domains are now shown in a comparable

position. Please note that the colouring scheme used in Fig. 1 and the rest of the MS is defined in the legend to Fig. 1. One protomer is colored, the other one is grey.

14. fig. 3C: in the left-most cartoon, the gray protomer seems to have one green residue which should be gray as well.

Thanks – has been fixed.

Reviewer #3 (Remarks to the Author):

The manuscript by Agustoni et al. addresses the activation mechanism of a small sensory histidine kinase LvrB, which serves as a master regulator in the pathogen Leptospira. LvrB only consists of two domains: the N-terminal Rec domain responds to an upstream phosphorylation signal, which in turn activates the C-terminal histidine kinase via auto-phosphorylation. Despite the relatively small size, the authors have obtained the cryoEM structures of the full-length LvrB in two distinct dimeric forms, representing the kinase-inactive and kinase-activated states, respectively. Based on structural comparisons, the authors propose that the kinase activation entails significant rearrangement in signaling helices involving the coiled-coil formation at the dimer interface and breaking of dimer symmetry. As intriguing as these findings are for structural studies of LvrB, this proposal is not necessarily groundbreaking for sensory histidine kinases. Several cryoEM and crystal structures have been reported for full-length HKs in dimeric forms, suggesting a general strategy of allostery shared among sensory histidine kinases, although the mechanistic details remain elusive. A major challenge is to figure out how the allosteric action propagates through a dimeric framework, for which LvrB perhaps represents the best minimalist model for structural dissection.

We thank the reviewer for their generally positive appraisal our work and many thought-provoking questions.

To this end, the work is deemed to fall short of expectation. Given ~2 millions of particles picked for LvrB prepared with and without BeF_3 (a phosphorylation mimic), the two structures presented in this work were reconstructed from <10% particles, which begs some questions regarding the rigor of data analysis.

LvrB exhibits significant orientation bias, as shown in Figures S3 and S5. The reason for this is that LvrB partitions at the air-water interface, yielding many side view orientations. During 3D reconstruction, it is normal that starting from the crude particle picking, only a small percentage of picked particles are ending up in the final result. Automated initial picking is very crude and it is common that 50-80% of particles are removed by manual selection of 2D and 3D classes during processing (Cameron, CJF et al. *Commun Biol* 7, 1421 (2024). https://doi.org/10.1038/s42003-024-07045-0).

Here are some examples of typical works in the field, with similar or even smaller number of particles in the final reconstruction:
Tang, X., Chang, S., Luo, Q. et al. Cryo-EM structures of lipopolysaccharide transporter LptB2FGC in lipopolysaccharide or AMP-PNP-bound states reveal its transport mechanism. Nat Commun 10, 4175 (2019). https://doi.org/10.1038/s41467-019-11977-1

3 data sets:

- Initial: 1.5 mil — final: 95k — 6.3%
- Initial: 1.7 mil — final: 149k — 8.7%
- Initial: 1.9 mil — final: 546k — 28.7%

Liu, X., Orenday Tapia, L., Deme, J.C. et al. A new paradigm for outer membrane protein biogenesis in the Bacteroidota. Nature 647, 479–487 (2025). https://doi.org/10.1038/s41586-025-09532-8

3 data sets:

- o Initial: 8.5 mil final: 55k 0.6%
- o Initial: 26.3 mil final: 96k 0.4%
- o Initial: 4.9 mil final: 534k 10.9%

Nakagawa, R., Hirano, H., Omura, S.N. et al. Cryo-EM structure of the transposon-associated TnpB enzyme. Nature 616, 390–397 (2023). https://doi.org/10.1038/s41586-023-05933-9

1 data set:

- o Initial: 2.1 mil. Final: 98k 4.6%

Are there additional states other than these two reported?

The reconstruction is unbiased and its output includes all significantly populated states. We have done an additional 3D variability analysis on the inactive and the activated structures. The result is that there is some local flexibility (Review-only-Figure 2) but no further states that would be significantly populated in the present data set.

How flexible or dynamic is the LvrB molecules in solution?

As described in Fig. 4A-C, NMR data show that in the activated state, one of the CA domains has increased flexibility. A full analysis of the dynamics of the entire protein including all its domains will require extensive assignments and advanced spectroscopic analysis, which can hopefully be achieved in future work.

Is there any population of LvrB particles that allude to the splayed conformational state revealed by crystallographic studies?

This splayed state is stabilized by crystal contacts that are clearly not present in solution. It is thus only minimally populated and not expected to be detected in the cryo-EM experiment. Accordingly, the cryo-EM analysis did not pick up such state from the solution ensemble.

Overall, the topic of this work is significant, and the results are interesting. However, its publication in Nat. Comm. may require revision of the dimeric model in the inactive state (see below) as well as additional work based on more inclusive and in-depth particle analysis based on the advanced algorithms such as 3DVA and 3DFlex of cryoSPARC.

Thanks for this suggestion. We have done a 3D variability analysis of the inactive and the activated states. These data show for the inactive state some variability in the Rec domains, and for the active state some variability in the DHp domain (Review-only-Figure 2). These are local variations that highlight LvrB as a dynamic molecule, but do not change the overall interpretation.

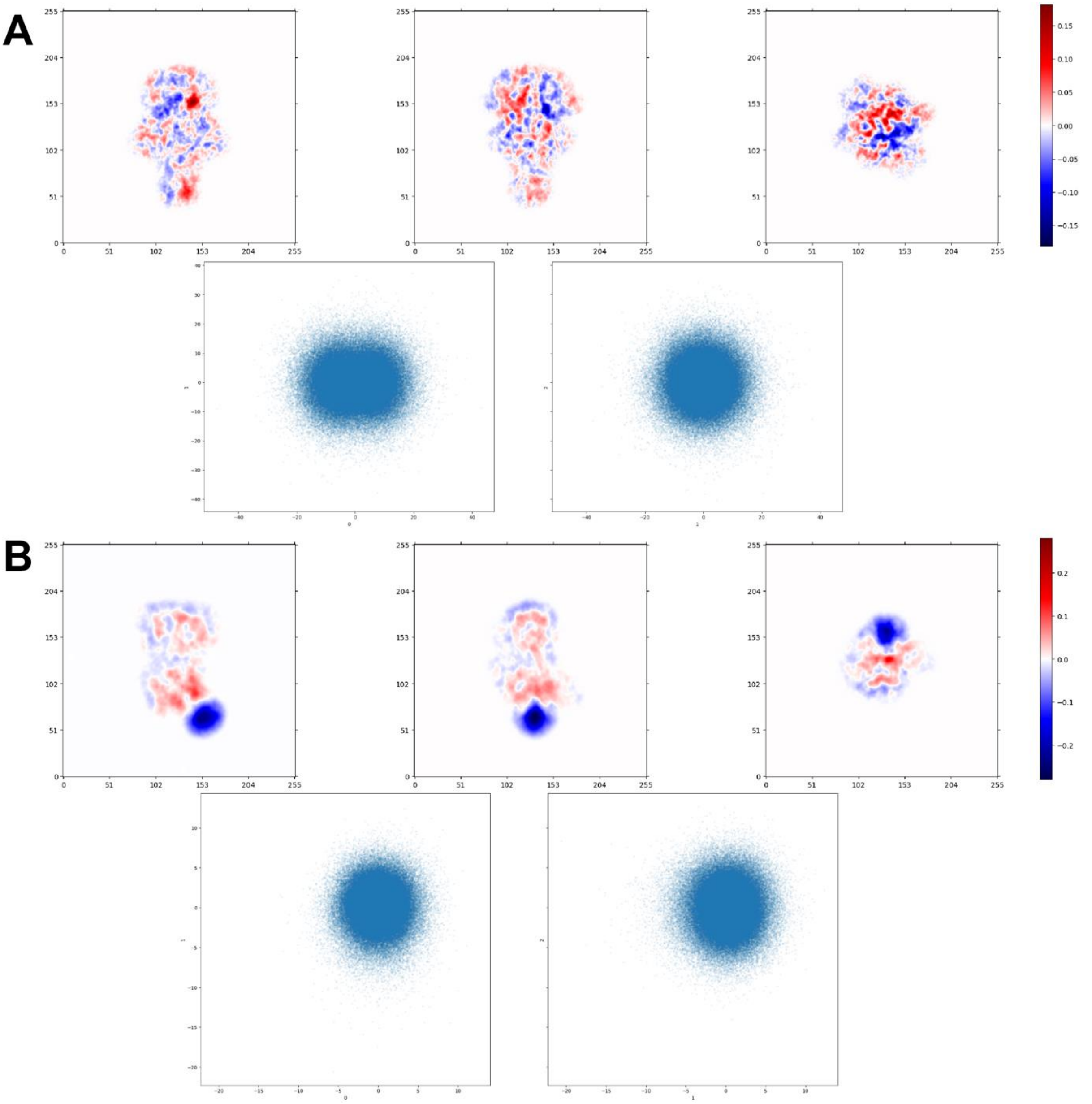

Review-only-Figure 2. Result of a 3D variability analysis of (A) inactive LvrB and (B) activated LvrB. Regions of local variability or shown in the top row, coherence of the ensemble is shown in the bottom row. The structures feature some local variability.

<u>MAJOR POINTS:</u>

1) Based on the view in Fig. 1D – the handedness of the inactive dimer is inconsistent between the cryo-EM map (left) and model (right). Specifically, the map clearly shows a dimer in a left-handed twist while the model shown in ribbon diagram seems to adopt a right-handed twist.

We thank the reviewer for spotting this issue. The gray linker connecting the DHp and CA domains, which passes in front of the red αS, had been hidden for clarity, thereby giving the impression of wrong handedness. We have now improved the Figure in the revised version of the manuscript.

2) Given the limited resolution of the reported cryoEM maps for both states (4.2Å and 5.9 Å, respectively), it is important to include a figure showing a side-by-side map comparison in the signaling helix regions as shown in Fig. 3c and Fig. S3H.

We are showing such figure now in Figure S9.

3) The proposal of the inverse Y-T switch in the REC domain is intriguing. Again, a side-by-side map comparison in the same region between two states would be informative.

The side chain orientations in the Rec domain are compared in Figure 2 and the Rec domains in different structures are compared in the new panel (F) within Figure S6

Does the inverse Y-T switch apply to both protomers? Is breaking of dimer symmetry also observed in the REC domain? In some REC-based sensory proteins, the Y-T switch may only take place in one of two promoters upon pseudo-phosphorylation (dimer asymmetry).

In the experimental conditions of the cryo-EM and X-ray structures, we worked with an excess of BeF_3^- and hence both Rec domains were pseudo-phosphorylated.

4) What is the mode of auto-phosphorylation for LvrB? trans or cis? Fig. 5 suggests a mode of cis phosphorylation. Is there any additional supporting (biochemical) data for this proposed cis- mode beyond the structural data? The information may help resolve possible ambiguity at the crossing of DHp and CA domains given the limited resolution of the activated state.

We thank the reviewer for raising this point. The mode of autophosphorylation of LvrB has not been experimentally resolved. Our proposal of cis-phosphorylation is based on the structural accessibility of the cis site and steric hindrance of the trans site in the activated state, consistent with reported examples in other HKs. We have clarified this reasoning in the revised Discussion.

5) Regarding the hemi-phosphorylation, the kinase activity assays presented in Fig. 1B and Fig. 1C do not seem to support this notion. While Fig. 1C (Phos-tag based) appears to render a 1:1 ratio in intensity between the phosphorylated (top) band and unphosphorylated (bottom) band, Fig. 1B (^{32}P based) shows a clear increase in the phosphorylated signal as a function of the reaction time while the total protein band of LvrB remains largely constant. Please explain.

Panel 1B depicts the rate at which LvrB undergoes self-phosphorylation in the presence of beryllium fluoride. As such, the top gel, which is not sensitive to phosphorylation, serves as a loading control for the bottom radiography blot which reports on the rate of LvrB's auto-kinase activity. The gel in panel C is a phosphorylation sensitive gel which captures both of LvrB's populations (i.e., (un)phosphorylated – hence the 2 bands.

Reviewer #4 (Remarks to the Author):
This manuscript presents the first 3D structures of both the activated and inactivated states of a full-length histidine kinase, LvrB, from Leptospira. LvrB is a Rec-controlled histidine kinase, a class of regulatory proteins central to bacterial two-component signaling systems. This study represents a significant advance, combining single-particle cryo-electron microscopy and X-ray crystallography to elucidate key structural transitions associated with LvrB activation. In the autokinase-inactive state, LvrB adopts a symmetric conformation, with unphosphorylated receiver (Rec) domains maintaining the separation of the αS helices. The authors also report a structure of the activated state, mimicked using BeF□$^{-}$, ADP, and Mg^{2+}. While the cryo-EM reconstruction is at relatively low resolution, a clear rearrangement of the Rec domains is observed, which is independently confirmed by well-resolved X-ray crystallographic data.

The resolution of both conformational states allows the authors to propose a mechanistic model of activation: Rec domain rearrangement leads to coiled-coil formation of the αS helices, release of the CA (catalytic and ATP-binding) domain, and ultimately, phosphorylation of LvrC, a newly identified downstream response regulator. These findings contribute important structural insight into a signaling pathway that has been largely unexplored at the molecular level. Overall, the study is technically rigorous, with comprehensive datasets and a well-supported interpretation. The methods are clearly described, and the integration of cryo-EM with crystallography is a notable strength. This work represents a substantial contribution to the field and advances our understanding of Rec-controlled histidine kinases and two-component systems. In conclusion, this is an excellent and impactful study, and I support its publication.

We thank the reviewer for the very positive assessment of our work and shared excitement.

Reviewer #5 (Remarks to the Author):

I co-reviewed this manuscript with one of the reviewers who provided the listed reports. This is part of the Nature Communications initiative to facilitate training in peer review and to provide appropriate recognition for Early Career Researchers who co-review manuscripts.

We thank the reviewer for their time and effort.

Reviewer #6 (Remarks to the Author):

I co-reviewed this manuscript with one of the reviewers who provided the listed reports. This is part of the Nature Communications initiative to facilitate training in peer review and to provide appropriate recognition for Early Career Researchers who co-review manuscripts.

We thank the reviewer for their time and effort.

Reviewer #1 (Remarks to the Author):

The authors have addressed the previous concerns. The manuscript has been significantly improved. However, a few minor technical points and formatting issues should be resolved prior to publication.

Figure 4: In panels C and D, please add direct labels or annotations to the figure itself to distinguish between the modelled and experimental structures. Relying solely on the figure caption for this distinction makes the data more difficult for the reader to parse quickly.

Thanks for pointing this out. This comment refers to Figure 3. We have added the labels.

Figure S14 & S15 (Stability Analysis): The RMSD plots indicate that certain replicas have not reached convergence (specifically Figure S14: Panel A, simulation #1 (inactive); Panel B, simulation #1 with TIP4P; and Figure S15: model 129-211, simulation #2). Given that three replicas were performed for each system, the authors should consider excluding these specific non-converged trajectories from the final calculated averages or downstream analysis to ensure the reported statistics reflect a stable equilibrium. At a minimum, the text should explicitly acknowledge these divergences and justify their inclusion.

We thank the reviewer for the careful assessment of the RMSD plots.

Figure S14A, simulation #1 (inactive). Visual inspection revealed that the increased RMSD value in the second half of the simulation stems from local unfolding of the last 9 residues of the artificially terminated helix α1. The local surroundings of Y87 and Y103 remained structurally compact and were not influenced. We thus kept the simulation and added a note to the caption to explain this.

Figure S15, model 129-211, simulation #2 indeed deviates from the other two replicas. We have excluded these data from the ensemble distance calculations in Figure S10A and added a note to that Figure caption.

Figure S14B, simulation #1 with TIP4P. Visual inspection revealed that the coiled-coil+Rec domains stay structurally stable, yet tilt dynamically relative to the DHp domain. At around 500ns, the RMSD drops, indicating its return to the initial structure. The temporarily increased RMSD is thus not indicative of destructive unfolding, but

rather captures the dynamic large-scale kinking of the coiled-coil/Rec domain relative to the DHp. This kinking movement is exactly the movement seen in other simulations, including those with TIP3P and for the shortened models. We have thus kept the simulation and added a note of explanation to the caption.

Line 675: Change "Refmac" to the standard nomenclature REFMAC5.

Done.

Reviewer #3 (Remarks to the Author):

The revised manuscript has largely addressed the concerns raised in the last round. It is well written with much improved clarity. In addition, the data is clearly presented, solid and comprehensive. While a few aspects of the mechanism remain unresolved, this work nevertheless represents a major advance on a highly interesting topic relevant to bacterial signaling. I support its publication in Nat. Comm. with minor changes. See below.

1) If possible, I suggest to present the crystal structure of LvrB in main figures in lieu of Fig. S6. Even though it is unknown at the moment how this “splayed” pseudo-phosphorylated dimer structure fits in the grand scheme of the LvrB activation, it represents a unique conformational state, perhaps an intermediate state trapped in crystal lattice as stated in Line 122-124. It is curious whether the third state in the proposed activation mechanism (Fig. 3C) is based on this crystal structure. To this end, it would be helpful for readers to gauge the tethering and handedness of the linker helices at the dimer interface if the two protomers are colored differently in Fig. S3B.

We fully agree and have added the X-ray structure to the new panel Figure 1F. We adapted its coloring to 1D and 1E for optimal comparison. In the structural transition shown in Fig. 3C, the crystal structure represents a form similar to the central models, where the two Rec domains have already separated but the coiled coil has not yet formed. This is now stated explicitly in the text.

2) The importance of E150 at the aS/DHp junction is noted. Yet, no mutational data on E150 is shown to support this. Any explanation?

Initially, E150 was one of many interesting candidates for functional probing. The mutant did not express, so it was not included in the manuscript. E150 then gained a lot of interest during the discussions with referees, and we did in silico mutagenesis as

requested by a referee, hence it now stands out. We are in the process of exploring alternative expression systems and hope to follow up with experiments in future work.

3) With a “liberated” (in other words “unresolved”) CA domain, one would take caution in assigning this asymmetric state to the “kinase-active” state. Based on Fig. 6, it is more compatible with a “phosphoryltransferase”-competent state. This circles back to my previous questions regarding the particle picking/filtering strategy. While it is indeed a common practice to use only a small fraction of particles (4% of total particles picked in Fig. S5; and <10% in Fig. S3) for 3D construction and refinement, it is not necessarily the best practice, particularly in this case where different conformational states of varying population are important for mapping out the trajectory of the kinase activation. An inclusive strategy that explores all possible states is critical. I also understood that such a task is beyond the scope of this work.

We agree with the referee. The activated state of LvrB as detected by cryo-EM is an ensemble of conformations with coherence in the Rec/αS/DHp/one-CA part. It may contain substates that are currently unresolved, and it will be highly worthwhile to make future progress in this direction. We have thus been careful to avoid premature assignments regarding autokinase vs phosphotransferase configurations of the activated state.
